# Regulation of acute myocardial infarction by CircTMCC1 through mitochondrial dysfunction and AMPK/mTOR-driven M1 macrophage polarization: role in QFR assessment

**DOI:** 10.1038/s41420-026-03213-9

**Published:** 2026-07-06

**Authors:** Mengmeng Ren, Shu He, Mengyang Duan, Boyu Chi, Zhiyuan Chen, Zhenyu Zhao, Zhongga Ciren, Yangzong Qiangba, Yahong Fu, Gaochao Wang, Liang Yuan, Fenghui An, Enzhi Jia

**Affiliations:** 1 Department of Cardiovascular Medicine, The First Affiliated Hospital of Nanjing Medical University, Nanjing, China; 2 Department of Cardiovascular Medicine, Lhasa People’s Hospital, Lhasa, China; 3 Changzhou Hospital to Nanjing University of Chinese Medicine, Changzhou, China; 4 Department of Cardiovascular Medicine, Chongqing Hospital of Jiangsu Province Hospital, Chongqing, China; 5 Department of Critical Care Medicine, The Friendship Hospital of Ili Kazakh Autonomous Prefecture, Yining, China

**Keywords:** Cell polarity, Gene regulation, Cardiovascular diseases

## Abstract

Circular RNAs (circRNAs) have been implicated in various cardiovascular diseases and hold promise as diagnostic biomarkers and therapeutic targets. However, the roles and mechanisms of circRNAs in coronary artery disease (CAD) and its severe complication, acute myocardial infarction (AMI), remain unclear. CircRNA sequencing, fluorescence in situ hybridization, and quantitative PCR were used to assess circTMCC1 expression in human coronary artery segments, peripheral blood mononuclear cells (PBMCs) from CAD patients, M1 macrophages, and an AMI mouse model. Multiple analytical methods were employed to investigate the predictive value of circTMCC1 for quantitative flow ratio (QFR) measurements. In vitro, we employed plasmid overexpression, small interfering RNA transfection, flow cytometry, immunofluorescence, reactive oxygen species (ROS), and mitochondrial membrane potential assays. In vivo, Masson’s trichrome, hematoxylin and eosin staining, and immunohistochemistry were performed. Mechanistic investigations included bioinformatics, RNA pull-down, RNA immunoprecipitation, co-immunoprecipitation, western blotting, and immunofluorescence. CircTMCC1 was significantly upregulated in CAD patients (*p* < 0.001) and associated with poor prognosis in AMI mouse models. CircTMCC1 was highly expressed in M1 macrophages (*p* < 0.001), and silencing its expression reduced M1 polarization, improved cardiac function after infarction, and regulated mitochondrial autophagy. Mechanistically, circTMCC1 facilitates the interaction between annexin A1 and the E3 ligase TRIM38, leading to annexin A1 degradation. Additionally, the AMPK/mTOR signaling pathway was identified as a downstream target of circTMCC1. These findings suggest that circTMCC1 may serve as a promising diagnostic biomarker and therapeutic target for CAD and AMI, potentially improving prognosis.

**Figure:**
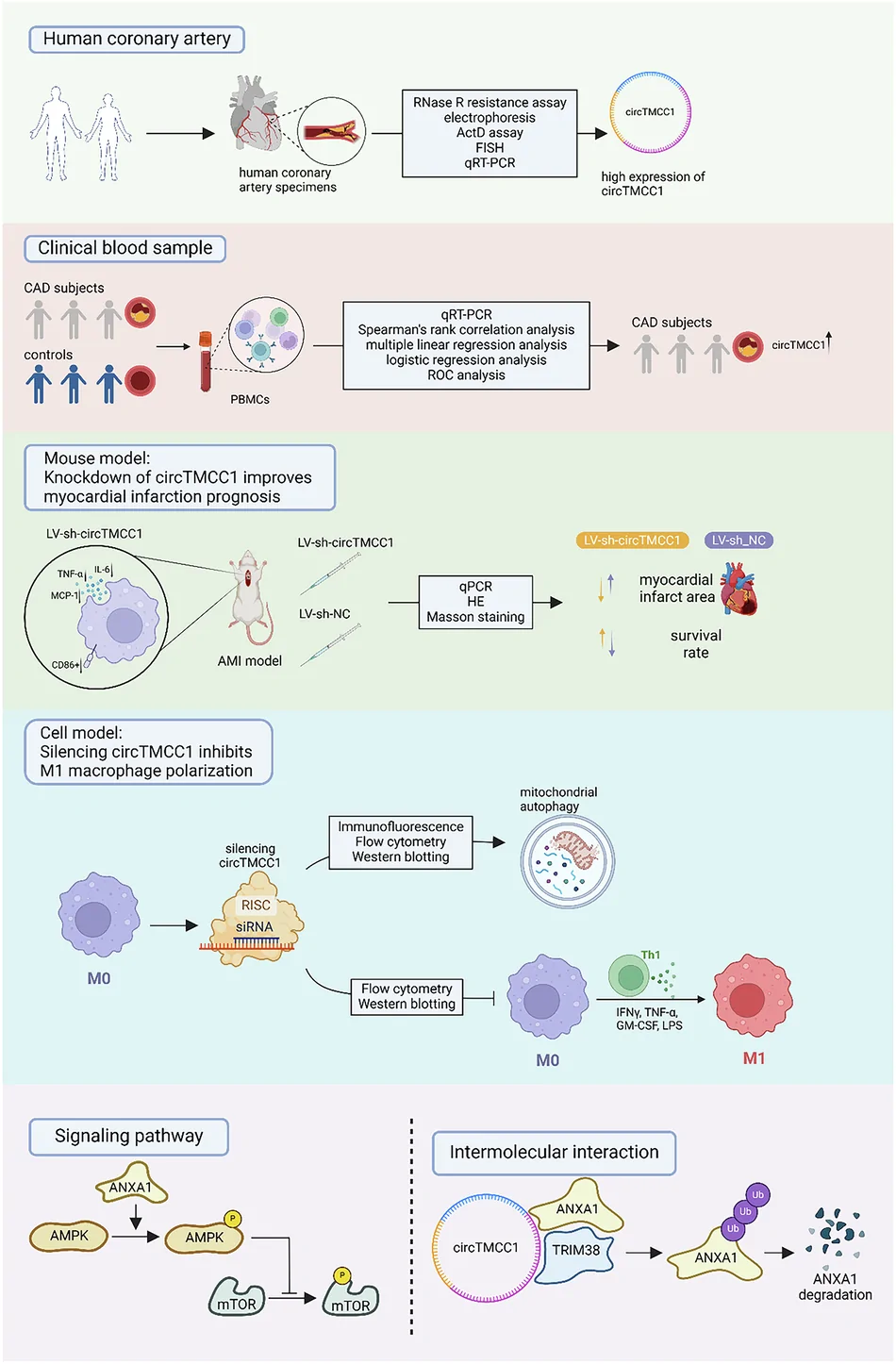

## Introduction

Cardiovascular disease (CVD), particularly ischemic heart disease (IHD), is a leading cause of death and health loss worldwide. In 2022, CVD caused approximately 19.8 million deaths, among which IHD—especially acute myocardial infarction (AMI)—had the highest age-standardized disability-adjusted life years (DALYs) at 2275.9 per 100,000 population [1, 2], highlighting the urgent need to identify diagnostic biomarkers and therapeutic targets for AMI.

Circular RNAs (circRNAs) are a class of non-coding RNAs (ncRNAs) characterized by a covalently closed loop structure, which confers high stability, sequence conservation, and resistance to degradation. circRNAs play important roles in cardiovascular diseases and represent potential diagnostic and therapeutic targets [3–7]. The primary pathological basis of AMI is atherosclerosis, which is characterized by inflammation, endothelial dysfunction, and lipid accumulation [8–10]. During the progression of atherosclerosis, myeloid cells can destabilize plaques and induce rupture [11]. Following AMI, myocardial tissue is infiltrated by neutrophils and monocyte-derived macrophages. Macrophages can polarize into pro-inflammatory M1 or anti-inflammatory M2 phenotypes, influencing inflammation resolution and tissue repair [12–15]. In addition, microvascular dysfunction following revascularization adversely affects long-term prognosis [16].

During the pathological process of AMI, cardiomyocytes subjected to ischemia and hypoxia exhibit mitochondrial oxidative phosphorylation dysfunction, leading to insufficient ATP synthesis. This, in turn, triggers a decline in mitochondrial membrane potential, calcium overload, and elevated ROS levels. These changes ultimately induce the opening of the mitochondrial permeability transition pore and rupture of the outer mitochondrial membrane, releasing pro-apoptotic factors (e.g., cytochrome c). Ischemia-reperfusion injury further exacerbates the above processes. Meanwhile, autophagy is markedly suppressed during AMI, impairing the clearance of damaged organelles and thereby aggravating myocardial injury [17, 18].

Annexin A1 (ANXA1) can regulate macrophage polarization through the FPR2/ALX-AMPK-mTOR pathway, inhibit vascular smooth muscle cell phenotypic switching, alleviate endothelial dysfunction, and protect against ischemia-reperfusion injury [19–23]; however, its precise role in AMI remains unclear. The tripartite motif-containing protein TRIM38 negatively regulates TLR3/4-mediated innate immune responses and interacts with MITA to reduce its K48-linked ubiquitination [24–26]. Additionally, studies in tumor cells have shown that ANXA1 inhibits Bnip3 expression via the FPR1 axis, thereby blocking mitophagy, suggesting that ANXA1 may have broader regulatory functions under stress conditions [21, 27].

In this study, we found that circTMCC1 is significantly upregulated in patients with coronary artery disease (CAD) and that its expression level correlates with poor prognosis in AMI mice. Further mechanistic investigations revealed that circTMCC1 promotes the interaction between ANXA1 and TRIM38, thereby influencing macrophage polarization and the inflammatory response in AMI. Knockdown of circTMCC1 reduces M1 macrophage polarization, suggesting that circTMCC1 holds promise as a therapeutic target for AMI.

## Results

### Identification and characterization of circTMCC1

**Fig. 1:**
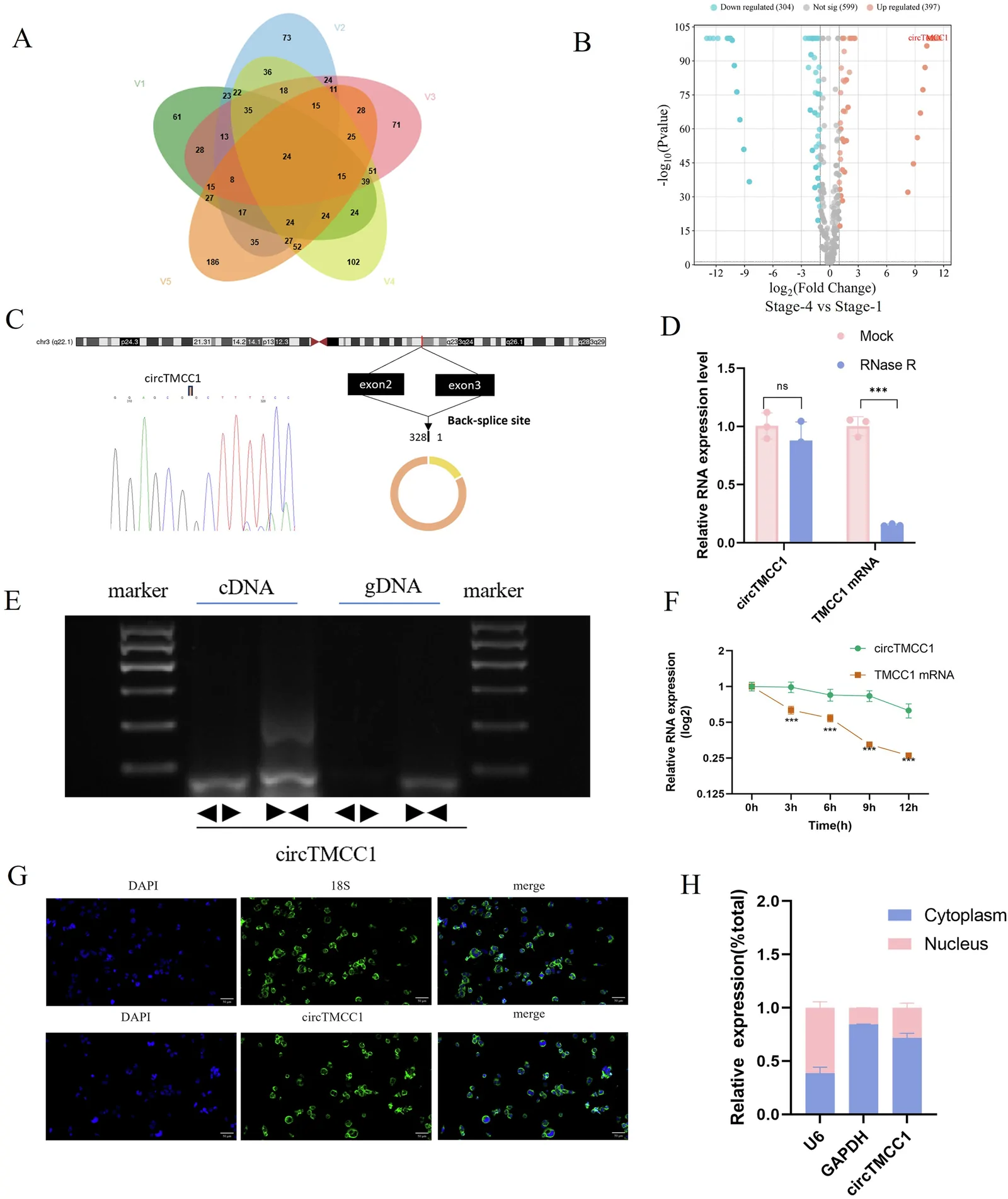
Identification and characterization of circTMCC1. **A** The Venn diagram shows the differential circRNA of lipid-striation stage grade 1 and fibrous plaque grade 4 in the sequencing data of coronary atherosclerosis specimens (V1–V5: Grouping of CircRNAs Highly Expressed in Sequenced Coronary Tissue Specimens With Atherosclerosis Grade 4 Compared With the Remaining 4 Lesser-Grade Lesions) (**B**) Volcano plot of screened differentially expressed circRNA. The stage grade was classified as follows: 1, stenosis from 0 to 25%; 2, stenosis from 26–50%; 3, stenosis from 51–75%; 4, stenosis from 76–100%. **C** circTMCC1(hsa_circ_0067323) is formed by circularization of exons 2–3 of the gene TMCC1, and the back-splicing junction site was verified by Sanger sequencing. **D** Quantitative real-time PCR analysis of circTMCC1 and linear TMCC1 in M0 macrophage treated with RNase R (*n* = 3). **E** Existence of hsa_circ_0067323 in M0 macrophage was verified by agarose gel electrophoresis. Divergent (circular) primers (◄ ►) amplified circRNAs in cDNA but not in genomic DNA (gDNA), convergent (linear) primers (►◄) amplified from both cDNA and gDNA. **F** After actinomycin D treatment, the mRNA stability of circTMCC1 and TMCC1 in M0 macrophage was determined by quantitative real-time PCR (*n* = 3). **G** RNA FISH for circTMCC1and nuclei were stained with DAPI (scale bars, 50 μm). Green shows circTMCC1; blue shows DAPI. **H** Expression levels of the cytoplasmic control (GAPDH), the nuclear control (U6), and circTMCC1 were determined by quantitative real-time PCR in the cytoplasmic and nuclear fractions of M0 macrophage(*n* = 3). Data are presented as the mean ± SD. Statistical significance was determined using Student’s t-test for two group’s comparison, and multiple t test analysis performs many t tests at once, with each test comparing two groups of data. *p < 0.05, **p < 0.01, ***p < 0.001, ****p < 0.0001, ns not significant.

Previous studies have established that atherosclerotic stages vary across coronary segments; specifically, the left main trunk (LM) exhibits stage 4 intimal involvement, whereas segments like LAD4M, LAD4P, LCX4D, RCA4P, and RCA4M remain at stage 1 (fatty streaks). Building on the aforementioned pathological staging, we identified five groups of circular RNAs (circRNAs) that were markedly upregulated in stage 4 lesions but downregulated in stage 1 lesions [28] (Supplementary Table S1). A total of 24 circRNAs with significantly elevated expression in stage 4 atherosclerosis (|log_2_^Fold Change^ | > 2) were identified from sequencing data of coronary atherosclerosis specimens (Fig. 1A). Differential expression analysis was performed between the LAD4P (stage 1) and LM4 (stage 4) groups, and the most significantly dysregulated circRNAs were identified (Supplementary Table S2). Notably, circTMCC1 (hsa_circ_0067323) exhibited the greatest absolute log_2_^Fold change^ among all dysregulated circRNAs. Moreover, these results suggest that the expression of circTMCC1 was significantly higher in advanced atherosclerosis than in early atherosclerosis (*p* = 1.65 × 10^–6)^ (Fig. 1B).

CircTMCC1, formed by the circularization of exons 2-3 of the TMCC1 gene, is located at chromosome 3: 129546645-129551669 according to the UCSC Genome Browser (http://genome.ucsc.edu/; Fig. 1C). The loop structure of circTMCC1 was confirmed using an RNase R resistance assay, demonstrating that RNase R degraded linear TMCC1 RNA but not circTMCC1 (Fig. 1D). The presence of circTMCC1 was further validated by 2% agarose gel electrophoresis (Fig. 1E). Actinomycin D (ActD) assays in the human monocytic leukemia cell line (THP-1) showed that circTMCC1 was resistant to ActD, indicating that circTMCC1 has greater stability and a longer half-life than linear TMCC1 (Fig. 1F). Consistently, fluorescence in situ hybridization (FISH) and quantitative real-time PCR (qRT-PCR) in THP-1 cells revealed that circTMCC1 was predominantly located in the cytoplasm (Fig. 1G, H). Using different cell lines, we identified that macrophages had the highest expression of circTMCC1 compared with cardiomyocytes, smooth muscle cells, and endothelial cells (Supplementary Fig. A).

### CircTMCC1 as an independent predictor and potential biomarker of coronary atherosclerosis

**Fig. 2:**
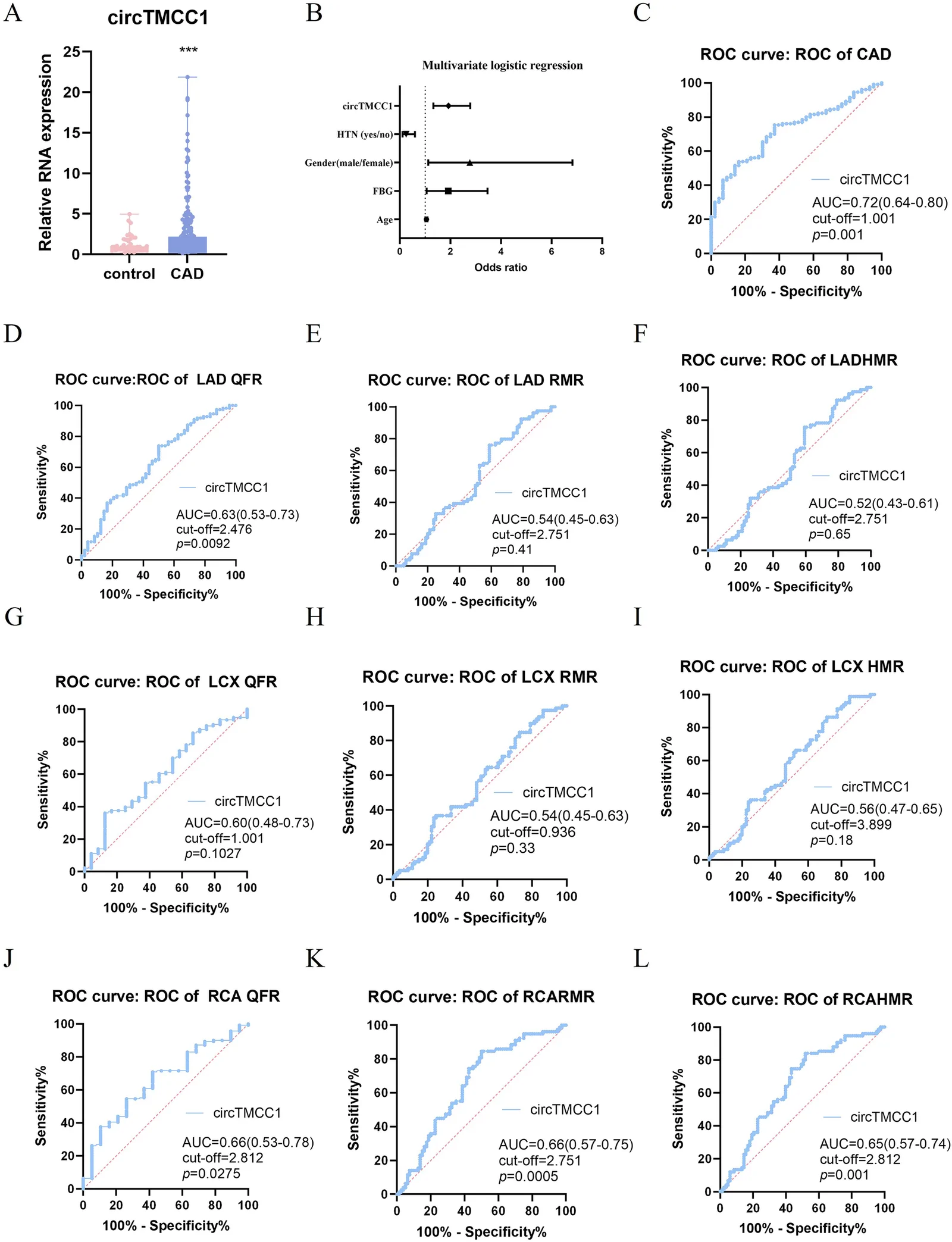
CircTMCC1 expression and diagnostic value in CAD. **A** CircTMCC1 was highly expressed in CAD (*n* = 130) compared with controls (*n* = 43) in the PBMCs. **B** Multivariate logistic of predictors of CAD prevalence in multivariate models. **C–L** ROC curve analysis of circTMCC1. Data are presented as the mean ± SD. Statistical significance was determined using Student’s *t* test for two groups’ comparison, one-way ANOVA followed by Tukey’s multiple comparisons test for multiple group comparisons. *p < 0.05, **p < 0.01, ***p < 0.001, ****p < 0.0001, ns not significant.

The relative expression levels of circTMCC1 in the peripheral blood mononuclear cells (PBMCs) from subjects with CAD and controls were detected by qRT-PCR. The relative expression level of circTMCC1 was significantly upregulated in the CAD group (*p* < 0.001) (Fig. 2A and Supplementary Table 3).

Logistic regression analysis was performed to identify independent predictors of CAD. Results of the univariate regression analysis are presented in Supplementary Table 4. Multivariable logistic regression analysis revealed that age, fasting blood glucose (FBG), gender, hypertension, and circTMCC1 were independent risk factors for CAD (Fig. 2B). The corresponding odds ratios (95% confidence interval) were 1.047 (1.006, 1.089) (*p* = 0.037), 1.911 (1.055–3.461) (*p* = 0.033), 2.765 (1.121–6.819) (*p* = 0.027), 0.248 (0.104–0.594) (*p* = 0.002), and 1.919 (1.325–2.779) (*p* = 0.001), respectively (Supplementary Table 5).

Based on previous trials, QFR ≤ 0.80 was considered hemodynamically significant [29]. Patients were stratified into two QFR cohorts: QFR1 (QFR ≤ 0.80) and QFR2 (QFR > 0.80). The median values for microvascular resistance were as follows: left anterior descending artery resistance microvascular resistance (LADRMR) 4.94 (IQR, 3.58–7.03) mm Hg*s/cm, LAD hyperemic microvascular resistance (LADHMR) 2.44 (IQR, 1.75–3.06) mm Hg*s/cm, left circumflex artery resistance microvascular resistance (LCXRMR) 6.98 (IQR, 5.26–8.86) mm Hg*s/cm, left circumflex artery hyperemic microvascular resistance (LCXHRM) 3.04 (IQR, 2.53–3.45) mm Hg*s/cm, right coronary artery resistance microvascular resistance (RCARMR) 5.34 (IQR, 4.25–6.83) mm Hg*s/cm, and right coronary artery hyperemic microvascular resistance (RCAHMR) 2.64 (IQR, 2.25–3.05) mm Hg*s/cm. These median values were used as cutoffs to assess the predictive value of circTMCC1 for coronary microvascular function.

To evaluate the diagnostic value of circTMCC1, receiver operator characteristic curve (*ROC*) analysis was conducted. As shown in Fig. 2C, the area under the *ROC* curve (*AUC*) for the circTMCC1 level in predicting CAD was 0.72(95% CI: 0.64–0.80, *p* < 0.001). In Fig. 2D-L, ROC analysis demonstrated that circTMCC1 predicted LAD-QFR with an AUC of 0.63 (95% CI: 0.53–0.73, *p* = 0.0092). The AUC for predicting RCA-QFR was 0.66 (95% CI: 0.53-0.78, *p* = 0.0275), for RCA-RMR was 0.66 (95% CI: 0.57–0.75, *p* = 0.0005), and for RCA-HMR was 0.65 (95% CI: 0.57–0.74, *p* = 0.001). Spearman correlation analysis revealed that circTMCC1 expression was negatively correlated with LADQFR (*r* = –0.223, *p* < 0.001), LCXQFR (*r* = –0.192, *p* = 0.015), RCAQFR (*r* = –0.279, *p* < 0.001), RCARMR (*r* = –0.250, *p* = 0.002), RCAHRM (*r* = –0.260, *p* = 0.001) (Supplementary Table 6).

**Fig. 3:**
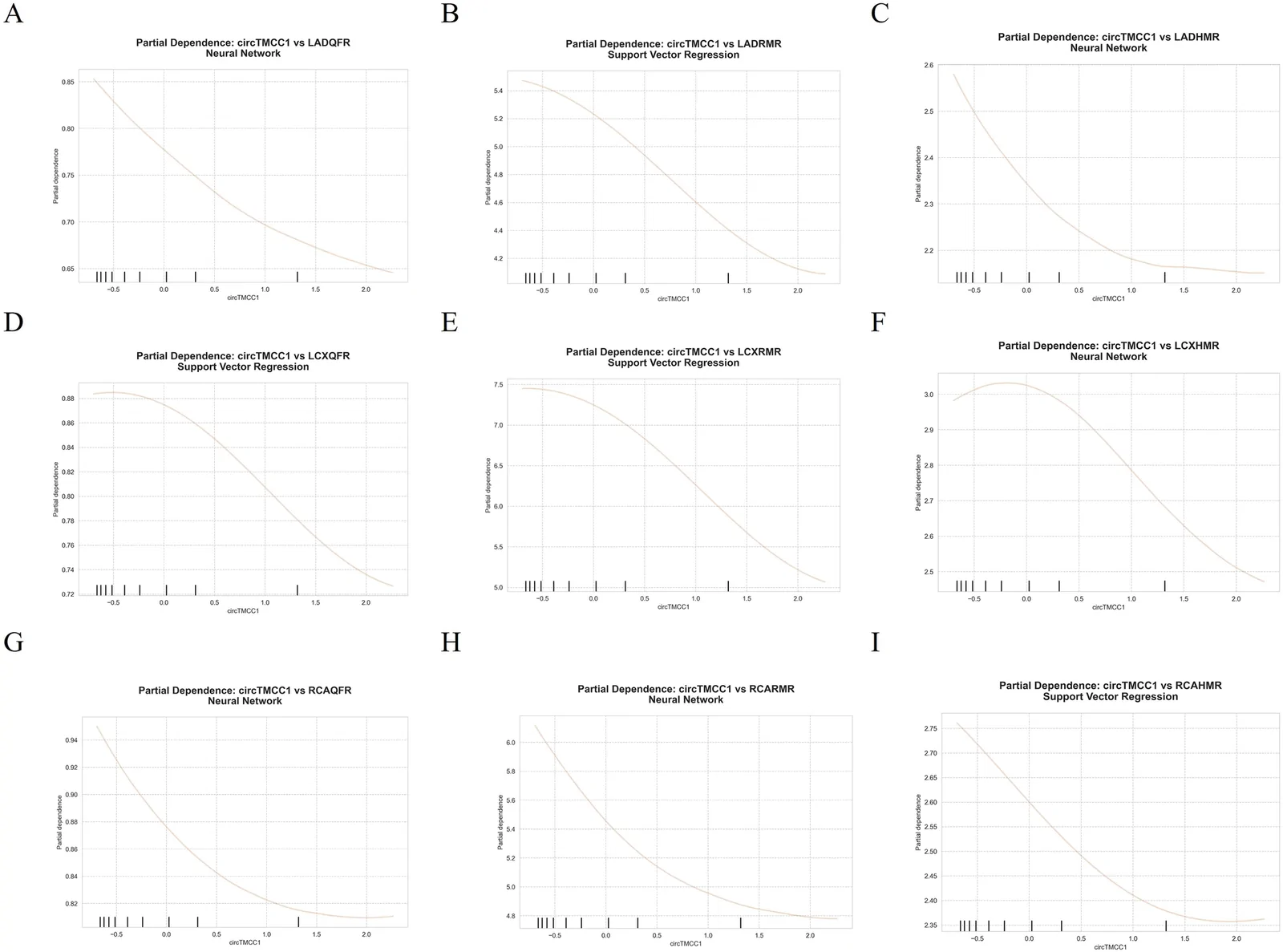
Partial dependence analysis of the relationship between circTMCC1 expression and coronary physiological parameters predicted by machine learning models. **A** Partial dependence plot illustrating the relationship between circTMCC1 and LADQFR as predicted by the neural network model. **B** Partial dependence plot showing the relationship between circTMCC1 and LADRMR as predicted by the support vector regression model. **C** Partial dependence plot depicting the relationship between circTMCC1 and LADHMR as predicted by the neural network model. **D** Partial dependence plot showing the relationship between circTMCC1 and LCXQFR as predicted by the support vector regression model. **E** Partial dependence plot showing the relationship between circTMCC1 and LCXRMR as predicted by the support vector regression model. **F** Partial dependence plot depicting the relationship between circTMCC1 and LCXHMR as predicted by the neural network model. **G** Partial dependence plot depicting the relationship between circTMCC1 and RCAQFR as predicted by the neural network model. **H** Partial dependence plot depicting the relationship between circTMCC1 and RCARMR as predicted by the neural network model. **I** Partial dependence plot showing the relationship between circTMCC1 and LCXHMR as predicted by the support vector regression model.

Among the 173 enrolled participants, 159 were eligible for LAD and RCA branch QFR analysis, while 160 were eligible for LCX branch QFR analysis. Machine learning models incorporating circTMCC1, age, lipid levels, blood urea nitrogen (BUN), pro-brain natriuretic peptide (pro-BNP), serum creatinine (Scr), and uric acid (UA) revealed a nonlinear negative correlation between circTMCC1 expression and QFR as well as coronary microvascular function (Fig. 3).

### Knockdown of circTMCC1 improves AMI prognosis

**Fig. 4:**
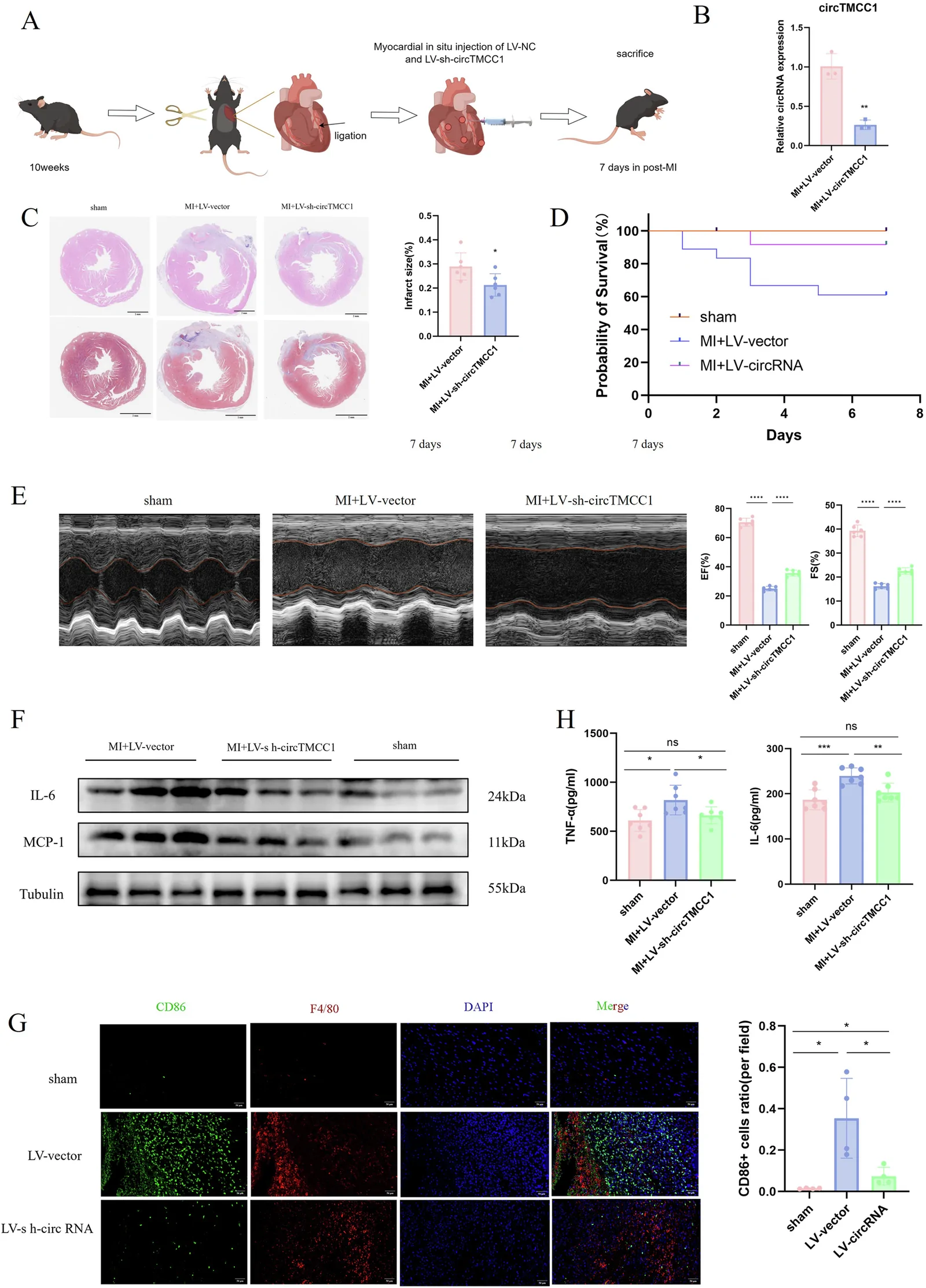
Silencing of circTMCC1 alleviates myocardial injury and inflammation after MI in mice. **A** The LAD of mice was ligation to construct a myocardial infarction model. **B** qPCR was used to verify the successful silencing of circTMCC1 in mice(*n* = 6). **C** Representative HE and Masson trichrome staining of cardiac tissue sections from the sham operation group and the LAD ligation groups (LV-vector and LV-sh-circTMCC1, *n* = 5 mice/group) at day 7 after MI, with quantification of infarct size (%) within the ischemic heart. Scale bar: 2 mm. **D** Survival rate of sham +LV-vector -operated(*n* = 10), LV-vector–treated(*n* = 18), and LV-sh-circRNA-treated mice(*n* = 12) after MI. **E** Representative M-mode echocardiograms obtained from sham, MI + LV-vector and MI + LV-sh-circTMCC1 (*n* = 5 mice/group) at day 7 after MI, and echocardiographic measurements of left ventricular ejection fraction and fractional shortening in the sham, MI + LV-vector, and MI + LV-sh-circTMCC1 groups (*n* = 5 mice/group) at day 7 after MI. **F** IL-6 and MCP-1 protein expressions at day 7 post-MI from sham+ LV-vector, MI + LV-vector and MI + LV-sh-circTMCC1 mice(*n* = 3). **G** Representative double-fluorescent immunostaining images of CD86 and F4/80. The positive CD86 (CD86^+^) cell areas were quantified (right) (*n* = 6). Scale bar:50 um. **H** plasma levels of TNF-α, IL-6 in sham+ LV-vector, MI + LV-vector and MI + LV-sh-circTMCC1 mice by ELISA assay (*n* = 7). Graphs depict mean ± SD. Statistical significance was determined using Student’s t-test for two group’s comparison, one-way ANOVA followed by Tukey’s multiple comparisons test for multiple group comparisons and two-way ANOVA followed by Bonferroni’s multiple comparisons test for comparison between different groups over time. The Kaplan-Meier method and the Log-rank (Mantel-Cox) Test were used to construct and analyze the survival curves. *p < 0.05, **p < 0.01, ***p < 0.001, ****p < 0.0001, ns not significant.

To investigate the function of circTMCC1 on macrophage inflammation after AMI, the mouse myocardium was injected with lentiviral vectors (LVs) carrying a control vector (LV-vector) or LV-sh-circTMCC1. An AMI model was established by ligating the LAD of the coronary artery in mice (Fig. 4A).

circTMCC1 was significantly upregulated in cardiac macrophages within the infarcted myocardium of mice, in contrast to normal myocardial tissue, where expression was undetectable (Supplementary Fig. B). qPCR was performed to ensure the successful silencing of circTMCC1 in the myocardium of mice (Fig. 4B). Notably, HE and Masson staining confirmed that LV-sh-circTMCC1 mice exhibited a lesser degree of myocardial infarct area compared with LV-sh-NC mice (Fig. 4C). These findings indicate that silencing circTMCC1 attenuates cardiac injury following AMI. Compared with MI mice treated with LV-sh-NC controls, those treated with LV-sh-circTMCC1 showed significantly improved survival, with 92% surviving compared with 61% in the control group (Fig. 4D). This survival benefit was accompanied by a marked improvement in cardiac function at 7 days after myocardial infarction (Fig. 4E).

To clarify the role of circTMCC1 on macrophage polarization, we analyzed the expression of macrophage phenotype markers in LV-sh-NC mice and LV-sh-circTMCC1 mice. Compared with LV-sh-NC mice, LV-sh-circTMCC1 mice exhibited significantly lower expression levels of IL-6 and MCP-1 (Fig. 4F), along with a marked reduction in the number of CD86⁺ cells. In contrast, CD86^+^ cells were increased in the MI group compared with the sham group (Fig. 4G). Moreover, transduction of macrophages with LV-sh-circTMCC1 significantly reduced the expression of M1 markers, including TNF-α and IL-6 (Fig. 4H). Consequently, these findings indicate that knockdown of circTMCC1 attenuates M1 macrophage polarization, suggesting a pro-inflammatory role of circTMCC1 in the post-AMI immune response.

### Silencing circTMCC1 inhibits M1 macrophage polarization and induces mitophagy in vitro

**Fig. 5:**
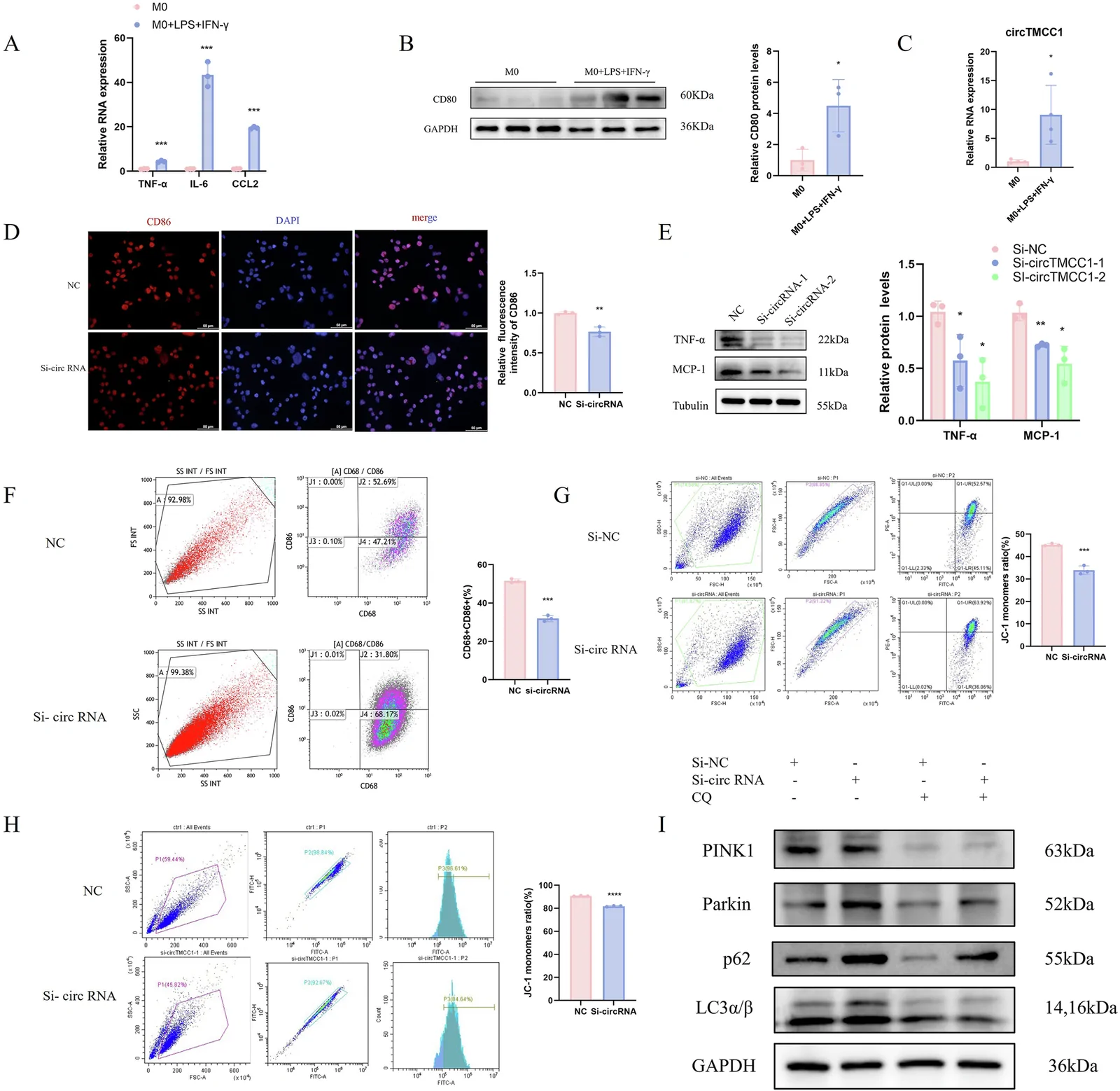
CircTMCC1 promotes M1 macrophage polarization and regulates mitochondrial function. **A** mRNA expression of M1 macrophage markers was studied by RT-qPCR and normalized by GAPDH expression. (*n* = 3). **B** Thp-1-derived M0 macrophages were stimulated with 100 nM LPS and 20 ng/mL IFN-γ, and the abundance of CD80 protein was detected by western blotting with specific antibodies (*n* = 3). GAPDH was used as the loading control. **C** The expression of circTMCC1 was detected in M1 macrophages. (*n* = 6) **D** Representative fluorescent immunostaining images of CD86 were quantified (right) (*n* = 3). Scale bar:50 um. **E** Western blot analysis showed that the protein levels of TNF-α and MCP-1 were significantly decreased upon silencing of circTMCC1 (*n* = 3). **F** The proportion of CD68 + CD86^+^ cells was detected by flow cytometry(*n* = 3). **G** Results of mitochondrial membrane potential were detected by flow cytometry(*n* = 3). The excitation/emission spectra of JC-1 monomers are 490 nm/530 nm, and those of JC-1 aggregates are 525 nm/590 nm. FITC-A: JC-1 monomers, PE-A: JC-1 aggregates. **H** Results of ROS were detected by flow cytometry(*n* = 3). **I** Cells transfected with si-circRNA were treated with 20μmol/L CQ for 36 h, and the PINK1, parkin, P62 and LC3 level in the indicated cells was measured by Western blotting. Data are presented as the mean ± SD. Statistical significance was determined using Student’s t-test for two groups’ comparison, one-way ANOVA followed by Tukey’s multiple comparisons test for multiple group comparisons. *p < 0.05, **p < 0.01, ***p < 0.001, ****p < 0.0001, ns not significant.

Given the critical role of macrophages in MI repair, we induced M1 macrophages (Fig. 5A, B) and assessed the expression of circTMCC1 in these cells (Fig. 5C). We then conducted loss-of-function studies to investigate the role of circTMCC1. Two siRNAs targeting the reverse splice junction of circTMCC1 significantly reduced its expression in vitro (Supplementary Fig. C). Subsequent immunofluorescence, flow cytometry, and western blotting analyses confirmed that silencing circTMCC1 led to decreased M1 macrophage polarization (Fig. 5D–F). We also found that circTMCC1 regulates mitochondrial autophagy in macrophages.

We preliminarily explored the potential impact of circTMCC1 on mitochondrial homeostasis in macrophages. Flow cytometry analysis showed that silencing circTMCC1 reduced intracellular reactive oxygen species (ROS) levels and improved mitochondrial membrane potential, as evidenced by a decreased JC-1 monomer ratio (Fig. 5G, H). In contrast, circTMCC1 overexpression produced the opposite effects, leading to elevated ROS accumulation and impaired mitochondrial membrane potential (Supplementary Fig. D, E). Western blot analysis revealed that after silencing circTMCC1, the levels of proteins associated with mitophagy initiation, PINK1 and Parkin, as well as the autophagosome marker protein LC3-II, were increased. However, simultaneous accumulation of the autophagy substrate p62 was observed (Fig. 5I). The accumulation of p62 suggests that the degradative phase of the autophagic flux may be affected. These preliminary observational data suggest that the loss of circTMCC1 may regulate macrophage polarization by modulating mitophagy and mitochondrial function.

### circTMCC1 directly interacted with protein ANXA1

**Fig. 6:**
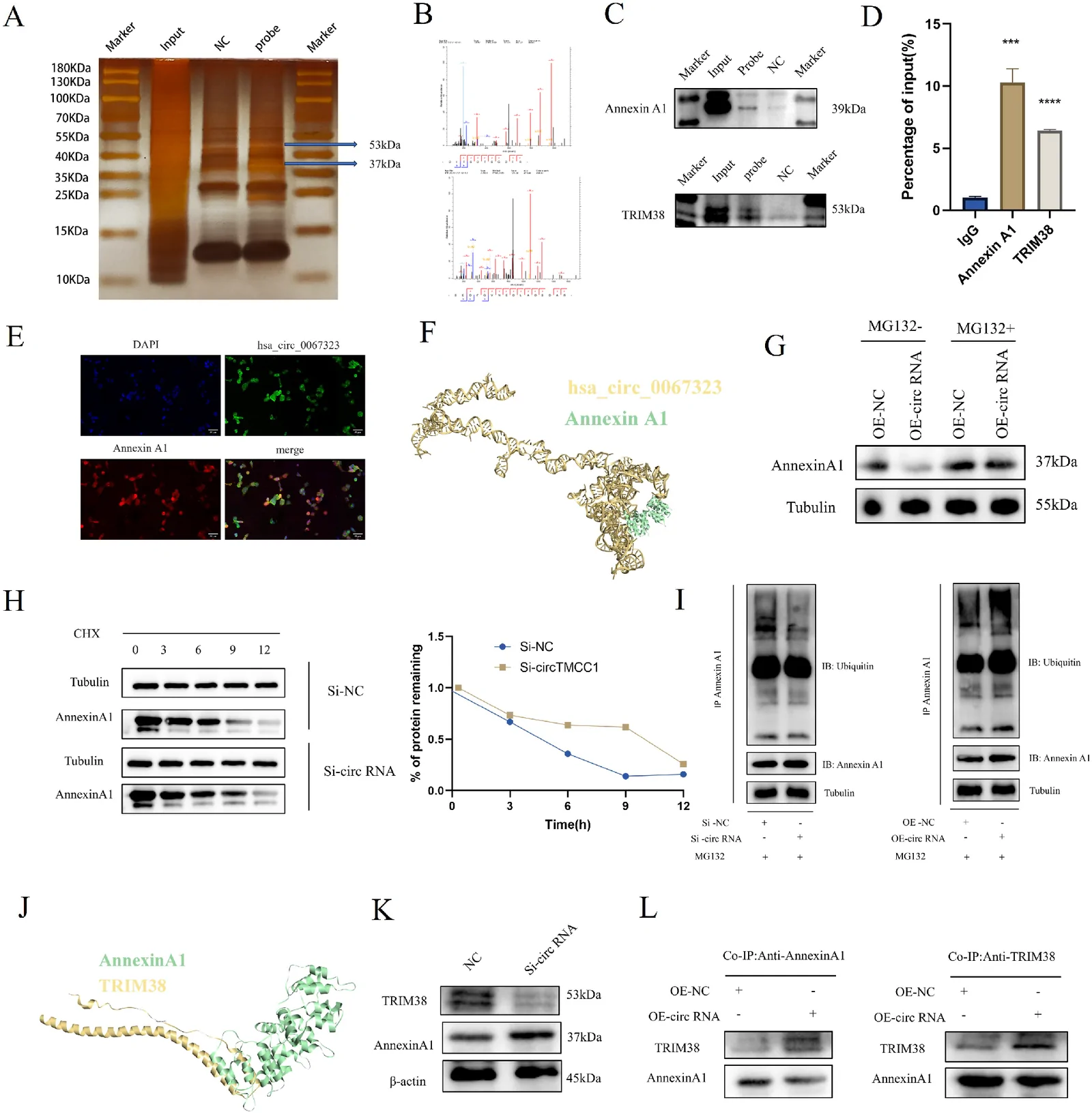
CircTMCC1 interacts with ANXA1 and regulates its protein stability via TRIM38. **A** Silver staining of the gel after SDS‒PAGE separation of the proteins that were immunoprecipitated with the 5’AFM biotin-labeled probe for circTMCC1 (probe) and the biotinylated antisense probe (NC) in RNA pull-down assays. The bottom arrow indicates the position of ANXA1, and the top arrow indicates the position of TRIM38. **B** Mass spectrometry analysis showed that ANXA1 was highly expressed in the circTMCC1 probe set according to peptide identification results (the up: NC group; the down: circTMCC1 probe group) **C** Western blot analysis following the RNA pull-down assay and **D** qRT‒PCR analysis following the RIP assay confirmed the interaction of circTMCC1 with ANXA1 and TRIM3 respectively (*n* = 3). **E** FISH showed that circTMCC1 was colocalized with ANXA1 in M1 macrophage. **F** 3-Dimensional structure of the circTMCC1- ANXA1 complex. **G** Western blotting analysis of ANXA1 protein levels regulated by circTMCC1 with or without MG132 treatment. **H** THP-1 cells-derived macrophage with circTMCC1 overexpression or knockdown were treated with cycloheximide (CHX) for the indicated times. Western blotting analysis (left) and statistical analysis (right) of ANXA1 levels upon CHX treatment are presented, with the level at 0 h as a control. **I** The lysate of circTMCC1 knockdown or overexpressed cells was immunoprecipitated (IP) with anti- ANXA1 antibodies and then used for western blot analysis of ubiquitin and ANXA1. **J** The interaction between ANXA1 and TRIM38 proteins was predicted by the HDOCK server. The predicted structure was visualized by Discovery Studio 4.5 Client. **K** The lysate of circTMCC1 overexpressed cells was immunoprecipitation (IP) with anti-TRIM38 antibody and anti-ANXA1 antibody respectively, and the binding effect of the two proteins was analyzed by western blot. **L** The expression levels of ANXA1 and TRIM38 proteins after knockdown or overexpression of circTMCC1. Data are presented as the mean ± SD. Statistical significance was determined using Student’s t-test for two group’s comparison. *p < 0.05, **p < 0.01, ***p < 0.001, ****p < 0.0001, ns not significant.

We next investigated the molecular mechanisms by which circTMCC1 promotes M1 macrophage polarization. To identify potential protein interactors of circTMCC1, we designed 5′FAM-biotin-labeled probes specific for circTMCC1 and a biotin-labeled antisense probe as a negative control (NC) for RNA pull-down assays. Silver staining revealed a distinct band at approximately 37 kDa in the probe group but not in the NC group (Fig. 6A). Mass spectrometry analysis of proteins near 37 kDa identified ANXA1 as the top candidate, based on both protein score and peptide coverage (Fig. 6B).

Immunoblotting with an anti-ANXA1 antibody confirmed that ANXA1 was specifically pulled down by the circTMCC1 probe (Fig. 6C). RNA immunoprecipitation (RIP) further demonstrated that circTMCC1 was significantly enriched in the ANXA1 precipitate relative to the IgG control (Fig. 6D). In addition, FISH revealed clear colocalization of circTMCC1 with ANXA1 in M1 macrophages (Fig. 6E). Collectively, these results demonstrate that ANXA1 is a direct binding partner of circTMCC1.

To predict the potential binding mode, molecular docking between circTMCC1 and ANXA1 was performed using the HDOCK server and visualized with Discovery Studio 4.5 Client (Fig. 6F).

### CircTMCC1 recruits E3 ligase TRIM38 to promote ANXA1 degradation

We next investigated whether circTMCC1 regulates ANXA1 expression. qPCR revealed that circTMCC1 overexpression or knockdown did not alter ANXA1 mRNA levels (Supplementary Fig. F), prompting us to examine post-transcriptional regulation.

To determine whether circTMCC1 affects ANXA1 protein stability, we treated cells with the proteasome inhibitor MG132. MG132 abolished the reduction in ANXA1 protein induced by circTMCC1 overexpression, indicating that circTMCC1 promotes proteasomal degradation of ANXA1 (Fig. 6G). Consistently, cycloheximide (CHX) chase assays demonstrated that circTMCC1 knockdown significantly prolonged the half-life of ANXA1 protein in M1 macrophages (Fig. 6H).

We next assessed the ubiquitination level of ANXA1 by co-immunoprecipitation. circTMCC1 overexpression markedly increased the ubiquitination of endogenous ANXA1, whereas circTMCC1 knockdown exerted the opposite effect (Fig. 6I). These results demonstrate that circTMCC1 promotes ANXA1 degradation via the ubiquitin–proteasome pathway. Putative ubiquitination sites of ANXA1 were predicted using the bioinformatics tools CPLM [30] and PhosphoSitePlus [31] (Supplementary Fig. G).

We next sought to determine which E3 ligases were involved in the regulatory effect of circTMCC1 on ANXA1 degradation. The silver staining results suggest that the probe group revealed a specific band at about 53 kDa (Fig. 6A). Meanwhile, the mass spectrometry (MS) results revealed that an E3 ligase TRIM38 with a molecular weight of 53 kDa was present in the precipitate pulled down by the circTMCC1 probe (Fig. 6B). Immunoblotting confirmed the interaction between circTMCC1 and TRIM38 (Fig. 6C). Molecular docking using the HDOCK server predicted a potential binding interface between TRIM38 and ANXA1 (Fig. 6J). Notably, circTMCC1 knockdown reduced TRIM38 protein levels while increasing ANXA1 expression (Fig. 6K), suggesting that circTMCC1 may stabilize TRIM38 or promote its expression.

Based on these findings, we hypothesized that circTMCC1 may serve as a scaffold to facilitate the interaction between TRIM38 and ANXA1, thereby promoting ANXA1 ubiquitination and degradation. To test this hypothesis, co-IP assays were performed in M1 macrophages overexpressing circTMCC1. The results showed that the association between TRIM38 and ANXA1 was markedly enhanced upon circTMCC1 overexpression (Fig. 6L), supporting a scaffold role for circTMCC1 in bridging TRIM38 and ANXA1.

### Overexpression of ANXA1 inhibits M1 polarization of macrophages and regulates mitophagy in vitro

**Fig. 7:**
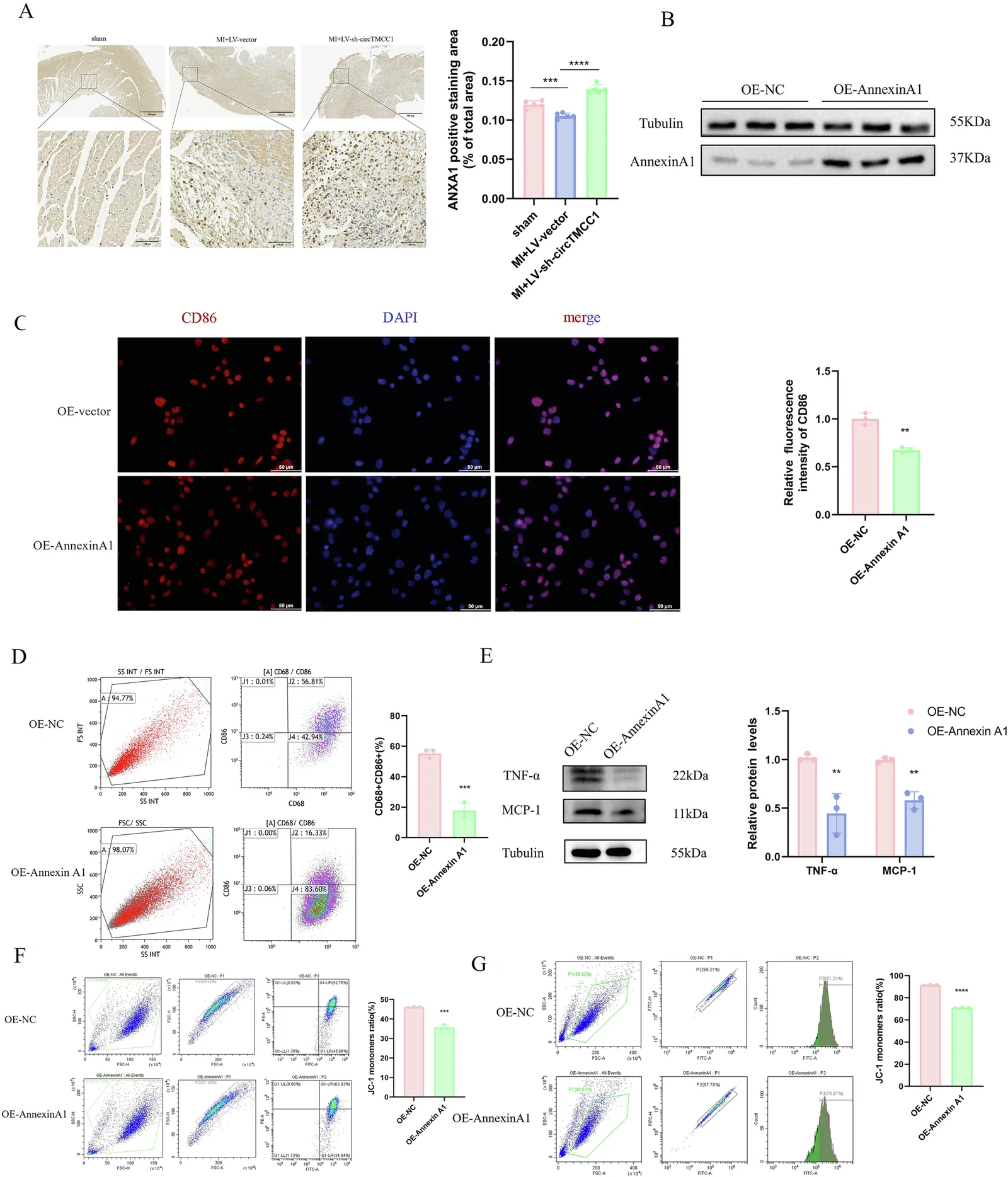
ANXA1 overexpression attenuates M1 macrophage polarization and mitochondrial dysfunction. **A** Representative IHC image of the target molecule in each group. The magnified areas were chosen to best depict the typical morphology of each group, though their exact locations may slightly vary. Quantitative analysis of the total positive area is depicted in the bar chart. (*n* = 5). Scale bar: 100 μm. **B** Efficiency of macrophage transfection with ANXA1 overexpression plasmid. **C** Representative fluorescent immunostaining images of CD86 were quantified (right)(*n* = 3). Scale bar:50 um. **D** The proportion of CD68 + CD86+ cells was detected by flow cytometry(*n* = 3). **E** Western blot analysis showed that the protein levels of TNF-α and MCP-1 were significantly decreased after overexpression of ANXA1 (*n* = 3) **F, G** Results of mitochondrial membrane potential and ROS were detected by flow cytometry (*n* = 3). Data are presented as the mean ± SD. Statistical significance was determined using Student’s t-test for two groups’ comparison, one-way ANOVA followed by Tukey’s multiple comparisons test for multiple group comparisons. *p < 0.05, **p < 0.01, ***p < 0.001, ****p < 0.0001, ns not significant.

To determine whether ANXA1 expression is regulated by post-infarction inflammation and circTMCC1, we performed immunohistochemical staining on mouse hearts following AMI. As shown in Fig. 7A, ANXA1 levels were significantly lower in the MI + LV-vector group than in the sham+LV-vector group, and this reduction was markedly reversed upon circTMCC1 knockdown. These results indicate that ANXA1 is downregulated during AMI and is subject to regulation by circTMCC1. In addition, we constructed an ANXA1 overexpression plasmid, and its efficiency was validated as shown in Fig. 7B. Immunofluorescence, flow cytometry, and western blot analyses confirmed that overexpression of ANXA1 significantly reduced M1 macrophage polarization (Fig. 7C–E). Moreover, flow cytometry showed that the proportion of ROS-positive cells and JC-1 monomers decreased significantly after transfection with OE-ANXA1 (Fig. 7F, G).

**Fig. 8:**
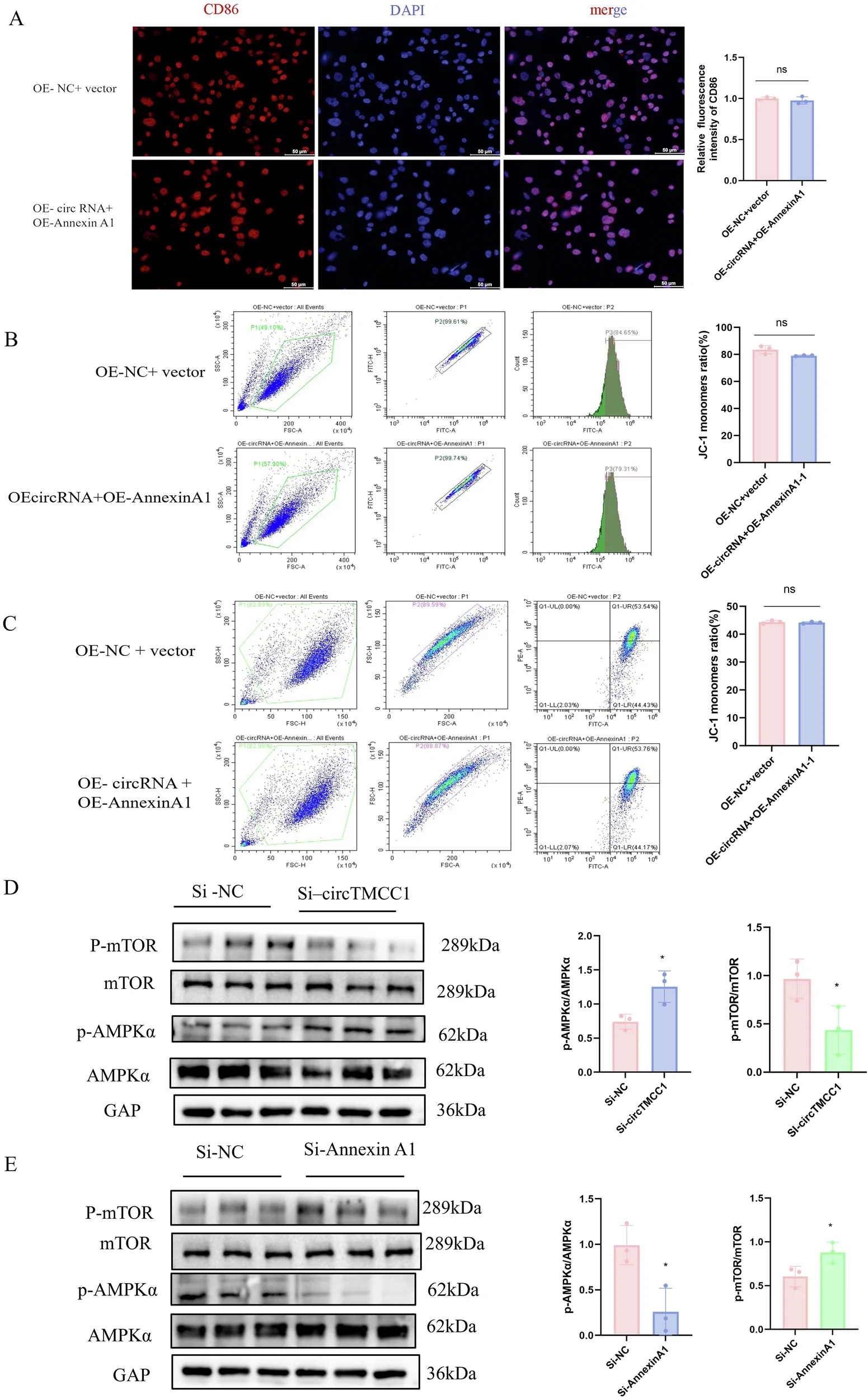
CircTMCC1 and ANXA1 regulate macrophage polarization and mitochondrial function via the AMPK/mTOR signaling pathway. **A** Representative fluorescent immunostaining images of CD86 were quantified (*n* = 3). Scale bar:50 um. **B, C** Results of mitochondrial membrane potential and ROS were detected by flow cytometry(*n* = 3). **D, E** Phosphorylation levels of AMPK, mTOR after knockdown of circTMCC1 or knockdown of annexin A1 (*n* = 3). Data are presented as the mean ± SD. Statistical significance was determined using Student’s t-test for the comparison of two groups. *p < 0.05, **p < 0.01, ***p < 0.001, ****p < 0.0001, ns not significant.

To further investigate the role of ANXA1 in the biological effects mediated by circTMCC1, both the circTMCC1 overexpression plasmid and the ANXA1 overexpression plasmid were co-transfected into M0 macrophages using a liposome-based transfection method, establishing a co-overexpression system for subsequent rescue experiments. Macrophages were transfected to overexpress circTMCC1 alone or in combination with ANXA1. As shown in Fig. 8A, circTMCC1 overexpression alone resulted in a significant increase in the expression of the M1 macrophage marker CD86 compared with control cells. When circTMCC1 and ANXA1 were co-overexpressed, CD86 expression was significantly reduced compared with circTMCC1 overexpression alone.

Mitochondrial membrane potential was subsequently assessed using JC-1 staining and flow cytometry. CircTMCC1 overexpression led to an increased proportion of JC-1 monomers, indicating a reduction in mitochondrial membrane potential (Fig. 8B, C). In contrast, co-overexpression of ANXA1 decreased the JC-1 monomer ratio compared with circTMCC1 overexpression alone, reflecting a partial restoration of mitochondrial membrane potential.

### CircTMCC1 promotes M1 macrophage polarization through ANXA1 and AMPK/mTOR pathway

To assess alterations in AMPK/mTOR signaling, the phosphorylation levels of AMPK and mTOR were examined in macrophages following circTMCC1 or ANXA1 manipulation. As shown in Fig. 8D, circTMCC1 knockdown resulted in a marked increase in AMPK phosphorylation, accompanied by a decrease in mTOR phosphorylation, compared with control cells.

In parallel, selective knockdown of ANXA1 was performed to evaluate its impact on the same signaling pathway. As shown in Fig. 8E, ANXA1 knockdown led to reduced AMPK phosphorylation and increased mTOR phosphorylation relative to control macrophages.

Direct comparison of these conditions revealed that circTMCC1 knockdown and ANXA1 knockdown exerted opposite effects on AMPK and mTOR phosphorylation under identical experimental settings.

## Discussion

Our study sheds light on the significant role of circTMCC1 in the progression of atherosclerosis, as demonstrated through coronary artery segment sequencing. Statistical analyses support the diagnostic value of circTMCC1 as a biomarker for CAD, particularly when combined with traditional risk factors. In both in vitro and in vivo experiments, we observed that circTMCC1 is highly expressed in M1 macrophages. Silencing circTMCC1 expression resulted in a significant reduction in M1 macrophage polarization and an improvement in cardiac function post-infarction. Mechanistically, we found that circTMCC1 functions as a scaffold, interacting with TRIM38 and ANXA1 to inhibit mitochondrial autophagy and the AMPK/mTOR signaling pathway, thereby influencing M1 macrophage polarization. Our findings suggest that circTMCC1 may serve as a promising prognostic biomarker and a potential therapeutic target for AMI.

Previous studies have confirmed that the QFR can be used to assess the occurrence of major adverse cardiac events (MACE) in patients with AMI [32, 33]. However, no studies have yet explored the relationship between RNA molecules, proteins, and QFR, or whether these factors can aid in assessing the prognosis of patients with AMI. In this study, we found that the expression of circTMCC1 may be non-linearly negatively correlated with QFR and coronary microvascular function, and could help predict coronary artery disease. However, our study has limitations, as we did not measure fractional flow reserve or the microcirculatory resistance index (IMR) to further assess myocardial perfusion. Future research will aim to address these shortcomings.

An interesting discrepancy arises when comparing our results to recent studies suggesting that circTMCC1 modulates inflammatory responses in astrocytes and inhibits the release of pro-inflammatory factors [34]. These variations in circTMCC1’s effects on inflammatory responses during AMI could be attributed to differences in circTMCC1 expression at various stages of AMI. This discrepancy highlights the need for further research to explore how circTMCC1’s role evolves over the course of AMI and in different tissue contexts. This intriguing phenomenon warrants additional investigation. Previous studies have shown that ANXA1 significantly reduces M1 macrophage inflammatory markers and activates the AMPK-mTOR pathway [35]. Our study introduces a novel perspective, positioning circTMCC1 as an upstream component of this regulatory network.

Ubiquitination is a critical post-translational modification of proteins [36]. Xu et al. demonstrated that circRNA-SORE binds the oncogenic protein YBX1 in the cytoplasm, preventing its interaction with the E3 ubiquitin ligase PRP19 and inhibiting PRP19-mediated degradation of YBX1 [37]. Similarly, circHIPK3 has been shown to act as a scaffold for the binding of p21 mRNA-binding protein HuR and the E3 ligase β-TrCP [38]. In our study, we demonstrate that circTMCC1 acts as a scaffold, facilitating the ubiquitination and degradation of ANXA1 by the E3 ligase TRIM38. Our data reveal new mechanisms by which ANXA1 regulates inflammatory responses following AMI, offering insights into the role of circRNAs in protein metabolism. The AMPK/mTOR signaling pathway is a classical intracellular signaling network whose dysregulation plays a pivotal role in the pathology of various cardiovascular diseases [39, 40]. This pathway, in conjunction with autophagy, helps fine-tune metabolic processes under stress [41], and it has been shown to reduce inflammation [42]. Growing evidence suggests that circRNAs can influence disease progression by modulating the AMPK/mTOR pathway [43, 44]. Moreover, ANXA1 has been shown to activate the AMPK/mTOR pathway in both brain ischemia/reperfusion (I/R) injury [21] and cardiac injury [35].

Several limitations of the present study should be acknowledged. First, although circTMCC1 expression was observed to be upregulated in inflammatory macrophages, the cellular origin of these macrophages could not be definitively determined. In particular, it remains unclear whether circTMCC1-expressing macrophages are predominantly derived from circulating monocytes or represent tissue-resident macrophage populations.

Second, the evaluation of mitophagy in this study was primarily observational. While changes in mitophagy-related markers were associated with circTMCC1 modulation, direct causal involvement of canonical mitophagy pathways, such as PINK1/Parkin signaling, was not experimentally assessed.

Third, although circTMCC1 expression was detected in human coronary atherosclerosis specimens, functional analyses were mainly performed in acute myocardial infarction animal models. Consequently, the role of circTMCC1 in chronic coronary atherosclerosis could not be directly examined.

Fourth, future studies using circTMCC1 overexpression in animal and cell models will be necessary to further validate its pro-atherogenic role and to fully establish gain-of-function evidence.

Finally, lentiviral vectors were used to manipulate circTMCC1 expression in vivo; however, this approach does not allow for precise cell-type-specific targeting. Therefore, the observed effects cannot be attributed exclusively to macrophages.

Future studies employing macrophage-specific genetic manipulation, bone marrow chimera models, and single-cell-based analyses will be necessary to address these limitations and to more precisely define the cell-type-specific role of circTMCC1 in post-infarction inflammation.

## Conclusion

Our study identifies circTMCC1 as a key regulator in atherosclerosis and AMI. Mechanistically, circTMCC1 acts as a scaffold to promote TRIM38-mediated ANXA1 degradation, thereby inhibiting mitophagy and the AMPK/mTOR pathway while promoting M1 macrophage polarization. Silencing circTMCC1 reduces M1 polarization and improves post-infarction cardiac function. Moreover, circTMCC1 exhibits strong diagnostic value for CAD, especially when combined with conventional risk factors. Thus, circTMCC1 may serve as a promising prognostic biomarker and therapeutic target for AMI.

## Materials and methods

### Autopsy sample collection, RNA extraction, and sequencing of coronary arteries

This study utilized coronary artery samples obtained from the autopsy of an 81-year-old man who died from a heart attack at Nanjing Medical University. Informed consent was obtained from the deceased’s family, and the autopsy was conducted in strict accordance with the university’s guidelines [28]. All experimental protocols were approved by the Ethics Committees of Nanjing Medical University and the First Affiliated Hospital of Nanjing Medical University. From the RNA sequencing of epicardial coronary arteries, 24 differentially expressed circRNAs were identified. These circRNAs may serve as diagnostic biomarkers or therapeutic targets in AMI.

### Study population

A total of 173 adult subjects who underwent coronary angiography at the First Affiliated Hospital of Nanjing Medical University from March 2023 to January 2024 were recruited in the present study. Detailed inclusion and exclusion criteria are described in our previously published article. They were all older than 18 years of age and had not undergone percutaneous coronary intervention or coronary artery bypass grafting before this hospitalization. All subjects underwent coronary artery angiography during this hospitalization. They were categorized into the coronary artery disease group (stenosis of the major epicardial coronary artery ≥50%) and the control group (stenosis of the major epicardial coronary artery < 50%) according to the American College of Cardiology/American Heart Association (ACC/AHA) classification. The exclusion criteria included congenital heart disease, rheumatic valvular disease, cardiomyopathy, cerebrovascular disease, malignant neoplasms, acute or chronic infectious diseases, adrenal dysfunction, severe liver dysfunction, severe kidney dysfunction, and thyroid dysfunction. Detailed clinical, demographic, hematologic, and angiographic examinations were performed for all the subjects enrolled in the present study [45]. The cumulative plaque burden was quantified using the Gensini score, which takes into account both the number of diseased coronary segments and the severity of stenosis [46]. The study was conducted in accordance with the Declaration of Helsinki and approved by the Ethics Committee of Nanjing Medical University and the First Affiliated Hospital of Nanjing Medical University (protocol code is 2021-SRFA-119 and date of approval is 24 February 2021). The patients or caretakers gave written informed consent to participate in this study as required by the Declaration of Helsinki.

### QFR and coronary microvascular function analysis

A certified operator, qualified to conduct QFR analysis, performed all measurements using QFR software (QFR® AngioPlus Galley Ⅱ), while a second, more experienced operator reviewed and made corrections to all analyses as needed. The results provided by the second operator were utilized for the analyses.

Angiographic images demonstrating optimal contrast opacification with clear visualization of all coronary branches were selected for QFR analysis. Additionally, the coronary microvascular function of the subjects was assessed. Resting Microcirculatory Resistance (RMR) and Simulated Hyperemic Microcirculatory Resistance (HMR) were calculated based on coronary angiography images.

### AMI mouse model

Murine AMI models were established by ligating the left anterior descending (LAD) artery. Briefly, ten-week-old male C57BL/6 mice were weighed and anesthetized with 1%-2% isoflurane inhalation, adjusted according to body weight. The mice were then intubated and ventilated using a small animal ventilator. A left thoracotomy was performed by making an incision between the third and fourth intercostal spaces, and the muscle and pericardium were dissected to expose the left anterior descending (LAD) artery. The LAD was ligated with a No. 8-0 silk suture 2 mm below the inferior border of the left atrial appendage, resulting in a pale anterior wall of the left ventricle. The chest was closed in layers with sutures. Sham+ LV-vector-operated mice underwent the same procedure without LAD ligation. On day 7 post-MI, blood was collected from the retro-orbital plexus for subsequent ELISA analysis, and cardiac tissues were collected after deep anesthesia before the mice were euthanized.

All procedures with animals were approved by the Institutional Animal Care and Use Committee of Nanjing Medical University (Approval No. IACUC-2404003) and performed in accordance with the guidelines from Directive 2010/63/EU of the European Parliament. After the study, all animals were anaesthetized by isoflurane inhalation (1–2%) and then euthanized by cervical dislocation.

### Cell culture and stimulation

The human monocytic cell line THP-1 was obtained from the cell bank of the Chinese Academy of Sciences (Shanghai, China). Prior to use, the cell lines were tested for mycoplasma contamination. THP-1 monocytes were cultured in RPMI 1640 medium (Gibco) supplemented with 10% fetal bovine serum (FBS; Vivacel) and 1% penicillin–streptomycin (Solarbio, China) at 37 °C in a 5% CO_2_ atmosphere. Differentiation into macrophages was induced by treating the cells with 100 ng/ml phorbol-12-myristate-13-acetate (PMA) (Sigma, USA) for 24 h. The macrophages were subsequently stimulated with human IFN-γ (Novoprotein, China, 20 ng/ml) and LPS (Sigma, USA, 100 ng/ml) for 48 h to polarize into M1 macrophages.

### Sanger sequencing

Sanger sequencing was carried out by TSINGKE Biological Technology Co., Ltd. (Beijing, China). Briefly, a 2 µL aliquot of the PCR product was electrophoresed on a 3% agarose gel to verify amplicon specificity. When a single clear band of the expected size was observed, membrane-based purification was used to remove primers and enzymes; when the band was unclear or multiple bands appeared, gel extraction was performed to recover the target fragment. Samples with excessive bands or low concentration were re-prepared. The purified DNA template was then subjected to Sanger sequencing, and the sequencing products were further purified to remove contaminants such as enzymes, fluorescent labels, and primers. Capillary electrophoresis was used for sequence resolution, with raw data interpreted by the sequencer’s built-in software. Sequence data were finally visualized and analyzed using Chromas software.

### Transfection of human THP-1 cells

Overexpression plasmids, small interfering RNA (siRNA) oligonucleotides, and corresponding negative controls were provided by GenePharma (Shanghai, China). THP-1 cells were differentiated for 24 h using 100 nM PMA. Subsequently, cells were transfected with 80 nM targeted siRNA or control siRNA using RNAFit (Hanbio, China). The specific siRNA sequences are listed in Supplementary Table 1. For plasmid transfections, the overexpression plasmid and its corresponding negative control were introduced using Lipofectamine 3000 Reagent (Invitrogen, Carlsbad, USA), following the manufacturer’s instructions.

### Fluorescence In situ hybridization (FISH) and subcellular fractionation

For RNA FISH experiments, cells were cultured on coverslips, fixed, permeabilized, and blocked before hybridization with FISH probes in hybridization buffer. Probes for circTMCC1 and 18S were synthesized by GenePharma (Shanghai, China). Cytoplasmic and nuclear RNA were isolated using the Cytoplasmic and Nuclear RNA Purification Kit (Norgen Biotek, Ontario, Canada). Cells were lysed on ice for 10 min with lysis buffer J, followed by centrifugation to remove cellular debris. RNA was then purified using appropriate spin columns. CircTMCC1 expression levels were quantified by qRT-PCR using GAPDH as a reference for cytoplasmic RNA and U6 for nuclear RNA.

### RNA isolation and quantitative real-time polymerase chain reaction (qRT-PCR)

Total RNA was extracted using Freezol Reagent (Vazyme China) and its concentration measured with a Nanodrop 2000 spectrophotometer (Thermo Fisher Scientific, Waltham, MA, USA). cDNA was synthesized using HiScript III All-in-One RT SuperMix (Vazyme, China). qRT-PCR was performed with ChamQ Universal SYBR qPCR Master Mix (Vazyme, China) on a 7500 Real-Time PCR system (Thermo Fisher Scientific, Waltham, MA, USA) for 40 amplification cycles. Primer sequences are listed in Supplementary Table 2.

### Western blotting

Total protein was extracted using RIPA buffer supplemented with phenylmethylsulfonyl fluoride and halt protease and phosphatase inhibitors. Protein concentrations were determined by BCA assay. Proteins were separated by SDS-PAGE and transferred to a 0.22 μm PVDF membrane. After blocking with 5% skimmed milk in tris-buffered saline for 2 h at room temperature, membranes were incubated overnight at 4 °C with primary antibodies (1:1000 dilution), followed by a 2-h incubation at room temperature with secondary antibodies (1:2000 dilution, A0208, Beyotime Biotechnology, China). Proteins were detected using an Enhanced ECL Chemiluminescence Kit (Vazyme China), and images were acquired with the Tanon 5200 Multi chemiluminescence system. Anti-GAPDH, anti-β-actin, anti-α-tubulin, anti-TNF-α, anti-IL-6, anti-MCP-1, anti-mTOR, and anti-p-mTOR antibodies were acquired from Proteintech (Wuhan, China). Anti-PINK1, anti-parkin, and anti-CD80 antibodies were obtained from ABclonal Technology (Wuhan, China). Anti-p-AMPK, anti-AMPK, anti-P62, and anti-LC3 antibodies were sourced from Wanlei Biotechnology Company (Shenyang, China).

### ELISA

Peripheral blood (2 mL) was collected from C57BL/6 mice, and serum was separated by centrifugation at 3500 rpm for 10 Min. The serum was aliquoted and stored at –80 °C until further analysis. Serum levels of IL-6 and TNF-α were measured using ELISA kits (Nanjing Yifeixue Biotechnology, China) according to the manufacturer’s protocols. The detection limits for IL-6 and TNF-α were 0.4-9 pg/mL and 20-400 pg/mL, respectively.

### Detection of reactive oxygen species (ROS)

Intracellular ROS levels were assessed by incubating cells with serum-free medium containing 10 μM DCFH-DA (Beyotime Biotechnology) for 20 min at 37 °C. After incubation, cells were washed three times with serum-free medium to remove excess DCFH-DA. The cells were then resuspended in PBS and analyzed using flow cytometry. Flow cytometric analysis was performed on a CytoFLEX Flow Cytometer (Beckman Coulter, USA), and data were processed with CytExpert software (https://www.beckman.com/flowcytometry/researchflowcytometers/cytoflex/software).

### Mitochondrial membrane potential (MMP) detection

For MMP assessment, 500,000 cells were mixed with 0.5 ml of cell culture medium and 0.5 ml of JC-1 dye solution (Beyotime Biotechnology, China) and incubated at 37 °C for 20 Min. Following incubation, the cells were washed three times with JC-1 staining buffer. Fluorescence intensity was measured using a CytoFLEX Flow Cytometer (Beckman Coulter, USA).

### RNA pull-down, silver staining, and mass spectrometry analysis

The biotin-labeled circTMCC1 probe and antisense were synthesized by GenePharma (Shanghai, China), and RNA pull-down assay was performed using a Pierce Magnetic RNA-Protein Pull-Down Kit (Thermo Fisher Scientific, Waltham, MA, USA). In brief, RNA probe (100 pmol) was mixed with 50 μL prewashed streptavidin magnetic beads and incubated for 30 min at room temperature with agitation. Then, the magnetic beads were washed with an equal volume of 20 mM Tris (pH 7.5), and 100 µL of 1X Protein-RNA Binding Buffer was added to the beads and mixed well. Subsequently, RNA-Protein Binding Reaction Master Mix was prepared and added to the RNA-bound beads, and the mixture was then incubated for 60 min at 4 °C with agitation. Finally, RNA-binding protein complexes were eluted with 50 µL of Elution Buffer and separated on SDS‒PAGE gels, and gels were then subjected to silver staining (Beyotime, Shanghai, China). The selected bands were identified by mass spectrometry (BiotechPack, Beijing, China) and Western blot analysis.

### RNA-binding protein immunoprecipitation (RIP) assays

Under the guidance of the manufacturer’s instructions, we performed RIP assays by using an EZ-Magna RIP Kit (Millipore, Billerica, USA). Briefly, 5 µg of the anti-annexin A1 antibody, anti-TRIM38 antibody and negative control IgG (Millipore, Billerica, USA) were incubated with magnetic beads for 30 min at room temperature with agitation. Then, the supernatant of the cell lysate was incubated overnight at 4 °C with antibody-conjugated magnetic beads for each RIP reaction. Finally, proteins in each sample were digested by proteinase K, and RNA was purified for subsequent downstream analysis.

### Masson’s trichrome and hematoxylin and eosin (HE) staining

Seven days post-MI, the mouse heart tissues collected after deep anesthesia were fixed in 4% paraformaldehyde. Cardiac tissues, excised from the region 1 mm below the ligature to the apex, were embedded in paraffin and sectioned at 5 μm thickness. The sections were dewaxed, hydrated, and stained with Masson’s Trichrome Staining Kit (Beyotime Biotechnology, China) and Hematoxylin and Eosin Staining Kit (Beyotime Biotechnology, China) according to the manufacturer’s instructions. Stained sections were examined and imaged using an Olympus CKX53 microscope (Tokyo, Japan).

### Immunofluorescence staining

Cells were fixed with Immunol Staining Fix Solution (Beyotime Biotechnology, China) for 10 min at room temperature, permeabilized with Immunostaining Permeabilization Buffer containing Triton X-100 (Beyotime Biotechnology, China) for 10 Min, and then blocked with 5% bovine serum albumin (BSA) for 30 Min. Cells were incubated overnight at 4 °C with a primary antibody against CD86 (1:2000 dilution, ABclonal Technology, China), followed by a 1-h incubation at room temperature with an FITC-conjugated secondary antibody. For cardiac tissue, paraffin sections were deparaffinized, rehydrated, subjected to antigen retrieval and blocking, and then incubated overnight at 4 °C with the primary antibody. Sections were subsequently incubated with a secondary antibody for 30 min at room temperature, developed with DAPI, and sealed with anti-fade mounting medium. Images were captured using a fluorescence microscope, and the percentage of positive cells and relative fluorescence intensity were analyzed with ImageJ.

### Immunohistochemical staining (IHC)

Immunohistochemical staining involved dewaxing, hydration, antigen retrieval, and blocking. Sections were incubated overnight at 4 °C with a primary antibody, followed by a 20-min incubation at 37 °C with a secondary antibody. 3,3’-diaminobenzidine was used for color development for 3 min, and sections were counterstained with hematoxylin, differentiated with hydrochloric acid-alcohol, and returned to blue with ammonia. After dehydration with anhydrous ethanol and clearing with xylene, sections were mounted in neutral resin and examined under a microscope.

### Co-immunoprecipitation (Co-IP)

Co-immunoprecipitation assays were conducted using a Protein A/G magnetic bead immunoprecipitation kit (Beyotime Biotechnology, China). Macrophages were lysed with inhibitor-containing lysis buffer, and the lysate was centrifuged to obtain the supernatant. Protein A/G magnetic beads were incubated with antibodies against ANXA1 and TRIM38 for 1 h at room temperature. The supernatants were then added to the antibody-bead complexes and incubated overnight at 4 °C with rotation. Proteins were eluted with SDS-PAGE Sample Loading Buffer (1X) and heated at 95 °C for 5 min, and analyzed by western blotting using antibodies against ANXA1, TRIM38, or ubiquitin (Proteintech, China).

### Statistical analysis

Statistical analyses were conducted using SPSS version 16.0 (IBM Corp, Armonk, NY) and GraphPad Prism 9 (GraphPad Software, San Diego, CA). Continuous variables are reported as mean ± standard deviation (SD) for normally distributed data and as median with interquartile range (IQR) for non-normally distributed data. Dichotomous variables are presented as counts and percentages (N1/N2). Comparisons between two groups were performed using the two-tailed Student’s *t*-test for normally distributed data and the Mann-Whitney *U* test for non-normally distributed data. For multiple group comparisons, One-way analysis of variance (*ANOVA*) was used for normally distributed data, and the Kruskal-Wallis test was applied for non-normally distributed data. Correlation analyses were conducted using Pearson’s correlation coefficient for normally distributed variables and Spearman’s rank correlation coefficient for non-normally distributed variables. All statistical tests were two-tailed, with a *p*-value of <0.05 considered statistically significant.

## Supplementary information

Supplementary Material

Supplementary Figure

Supplementary Figure

Supplementary tables

Supplementary Table 1

Supplementary Table 2

Supplementary tables 3

Supplementary tables 4

Supplementary tables 5

Supplementary tables 6

## Data Availability

The datasets and materials used in the study are available from the corresponding author.
