# Supplementary material for: Regulation of acute myocardial infarction by CircTMCC1 through mitochondrial dysfunction and AMPK/mTOR-driven M1 macrophage polarization: role in QFR assessment
Source: Cell Death Discov. 2026 Jul 6;12:386. doi: 10.1038/s41420-026-03213-9 (PMC13620124; doi:10.1038/s41420-026-03213-9)
Supplement: Supplementary file 1 [file 41420_2026_3213_MOESM10_ESM.docx]

Supplementary Table6. Correlations between circTMCC1 and characteristics of the study population: results of Spearman analyses.

| Variables | Relationship coefficient | *p*-value |
| --- | --- | --- |
| Age (years) | 0.024 | 0.753 |
| TC (mmol/L) | -0.046 | 0.549 |
| TG (mmol/L) | 0.045 | 0.558 |
| HDL-C (mmol/L) | -0.035 | 0.651 |
| LDL-C (mmol/L) | -0.025 | 0.745 |
| Bun (mmol/L) | -0.082 | 0.287 |
| Ua (μmol/L) | -0.071 | 0.363 |
| Scr (μmol/L) | -0.066 | 0.391 |
| Nt-pro-BNP (pg/mL) | 0.133 | 0.126 |
| LADQFR | -0.223 | <0.001 |
| LADRMR (mmHg·s/cm) | -0.109 | 0.170 |
| LADHMR (mmHg·s/cm) | -0.127 | 0.110 |
| LCXQFR | -0.192 | 0.015 |
| LCXRMR (mmHg·s/cm) | -0.115 | 0.146 |
| LCXHMR (mmHg·s/cm) | -0.131 | 0.098 |
| RCAQFR | -0.279 | <0.001 |
| RCARMR (mmHg·s/cm) | -0.250 | 0.002 |
| RCAHMR (mmHg·s/cm) | -0.260 | 0.001 |

TC: Total cholesterol; TG: Triglyceride; HDL-C: High-density lipoprotein cholesterol; LDL-C: Low-density lipoprotein cholesterol; Bun: blood urea nitrogen; Ua: Uric Acid; Scr: serum creatinine; Nt-pro-BNP: N-terminal pro-brain natriuretic peptide; LAD: left anterior descending artery; LCX: left circumflex artery; RCA: right coronary artery; QFR: Quantitative Flow Ratio; RMR: Resting Microvascular Resistance; HMR: Simulated Hyperemic Microvascular Resistance. The association of circTMCC1 with Age, TC, TG, HDL, LDL, FBG, and Gensini score, in 173 adult patients (CAD group=130, control group=43). Correlation coefficients are reported as Spearman correlations.
