# Supplementary material for: Regulation of acute myocardial infarction by CircTMCC1 through mitochondrial dysfunction and AMPK/mTOR-driven M1 macrophage polarization: role in QFR assessment
Source: Cell Death Discov. 2026 Jul 6;12:386. doi: 10.1038/s41420-026-03213-9 (PMC13620124; doi:10.1038/s41420-026-03213-9)
Supplement: Supplementary file 2 [file 41420_2026_3213_MOESM1_ESM.docx]

Figure4F

Sham+LV-vector

MI+LV-sh-circTMCC1

MI+LV-vector

**Tubulin**

35KDa

40KDa

55KDa

70KDa

15KDa

25KDa

**IL-6**

15KDa

25KDa

35KDa

**MCP-1**

Figure5B

M0

M0+LPS+IFN-y

55KDa

70KDa

**CD80**

35KDa

40KDa

**GAPDH**

Figure5E

Si-circTMCC1-1

Si-circTMCC1-1

NC

40KDa

55KDa

70KDa

**Tubulin**

35KDa

15KDa

25KDa

**MCP-1**

**TNF-A**

Si-circTMCC1-2

Si-circTMCC1-2

Si-circTMCC1-1

NC

Si-circTMCC1-1

NC

**Tubulin**

**Tubulin**

55KDa

70KDa

**TNF-A**

**TNF-A**

25KDa

35KDa

**MCP-1**

**MCP-1**

15KDa

Figure 5I

**PINK1**

**Parkin**

**P62**

LC3α/b /GAPDH

**LC3α/β**

**LC3α/β**

**LC3α/β**

15KDa

**GAPDH**

**GAPDH**

35KDa

**GAPDH**

P62/GAPDH

**PINK1**

**PINK1**

40KDa

55KDa

70KDa

**PINK1**

**GAPDH**

**GAPDH**

25KDa

35KDa

**GAPDH**

Parkin/GAPDH

**Parkin**

**Parkin**

55KDa

70KDa

**Parkin**

**GAPDH**

**GAPDH**

25KDa

40KDa

35KDa

**GAPDH**

**P62**

**P62**

**P62**

40KDa

55KDa

70KDa

**GAPDH**

**GAPDH**

15KDa

25KDa

35KDa

**GAPDH**

Fig6C (annexin A1)

70KDa

25KDa

35KDa

40KDa

55KDa

Fig6C (TRIM38)

70KDa

35KDa

40KDa

55KDa

Figure 6G

25KDa

35KDa

40KDa

55KDa

70KDa

**ANXA1**

**TUBULIN**

**TUBULIN**

**TUBULIN**

40KDa

55KDa

70KDa

**TUBULIN**

**ANXA1**

**ANXA1**

25KDa

35KDa

**ANXA1**

Figure 6H

Si-circRNA

Si-NC

55KDa

70KDa

**TUBULIN**

**TUBULIN**

25KDa

35KDa

40KDa

**ANXA1**

**ANXA1**

Si-circRNA

Si-NC

40KDa

55KDa

70KDa

**Tubulin**

**Tubulin**

25KDa

35KDa

**ANXA1**

**ANXA1**

55KDa

70KDa

**Tubulin**

**Tubulin**

40KDa

35KDa

**ANXA1**

**ANXA1**

Figure 5I-left

25KDa

35KDa

40KDa

55KDa

70KDa

**ANXA1**

**TUBULIN**

55KDa

40KDa

35KDa

25KDa

70KDa

**Ub**

Figure 5I - right

70KDa

25KDa

35KDa

40KDa

55KDa

**ANXA1**

**TUBULIN**

35KDa

25KDa

70KDa

40KDa

55KDa

**Ub**

Figure 6K (TRIM38)

55KDa

35KDa

40KDa

70KDa

Figure 6K (annexin A1)

35KDa

25KDa

40KDa

55KDa

70KDa

Figure 6K (β-Actin)

35KDa

25KDa

40KDa

70KDa

55KDa

Figure 6L-left (TRIM38)

70KDa

55KDa

40KDa

35KDa

25KDa

Figure 6L-left (annexin A1)

70KDa

25KDa

35KDa

55KDa

40KDa

Figure 6L-right(TRIM38)

25KDa

35KDa

55KDa

70KDa

40KDa

Figure 6L-right(annexin A1)

35KDa

40KDa

70KDa

55KDa

25KDa

Figure 7B`

OE-ANXA1

OE-NC

55KDa

70KDa

**TUBULIN**

40KDa

35KDa

25KDa

**ANXA1**

Figure 7E

40KDa

55KDa

70KDa

**TUBULIN**

25KDa

35KDa

**TNF-α**

15KDa

**MCP-1**

40KDa

55KDa

70KDa

**TUBULIN**

25KDa

35KDa

**ANXA1**

15KDa

**MCP-1**

Figure 8D (P-mTOR)

165KDa

210KDa

Figure 8D (mTOR)

320KDa

165KDa

270KDa

210KDa

Figure 8D (AMPKα)

70KDa

55KDa

Figure 8D (P-AMPKα)

55KDa

70KDa

100KDa

Figure 8D (GAPDH)

40KDa

35KDa

Figure 8E (P-mTOR)

165KDa

210KDa

Figure 8E (mTOR)

320KDa

165KDa

210KDa

270KDa

Figure 8E (AMPKα)

70KDa

55KDa

Figure 8E (p-AMPKα)

55KDa

70KDa

Figure 8E (GAPDH)

25KDa

15KDa

35KDa

40KDa
