# Supplementary material for: Regulation of acute myocardial infarction by CircTMCC1 through mitochondrial dysfunction and AMPK/mTOR-driven M1 macrophage polarization: role in QFR assessment
Source: Cell Death Discov. 2026 Jul 6;12:386. doi: 10.1038/s41420-026-03213-9 (PMC13620124; doi:10.1038/s41420-026-03213-9)
Supplement: Supplementary file 3 [file 41420_2026_3213_MOESM2_ESM.docx]

**Supplementary Figure**

(A)q-PCR analyses of circTMCC1 expression in macrophage (THP-1+PMA), AC16 human cardiomyocytes, human coronary artery smooth muscle cells and human coronary artery endothelial cells, respectively (n=5).

(B) Representative double-fluorescent immunostaining images of circTMCC1 and F4/80 in the sham group and MI group, along with quantitative analysis. Scale bar: 50 μm.

(C) Knockdown efficiency of two siRNA targeting circTMCC1 reverse splicing(n=3). Data are presented as the mean ± SD. Statistical significance was determined using Student’s t-test for two group’s comparison.

(D) The proportion of CD68+CD86+ cells was detected by flow cytometry (n=3)

(E) Results of mitochondrial membrane potential was detected by flow cytometry(n=3). The excitation/emission spectra of JC-1 monomers are 490nm/530nm, and those of JC-1 aggregates are 525nm/590nm. FITC-A: JC-1 monomers, PE-A: JC-1 aggregates.

(F) circTMCC1 overexpression or knockdown did not change the expression level of annexin A1 mRNA(n=3). Data are presented as the mean ± SD. Statistical significance was determined using Student’s t-test for two group’s comparison.

(G) The ubiquitination site of protein annexin A1 was predicted using CPLM and PhosphoSitePlus and then visualised using Discovery Studio 4.5 Clien. **p* < 0.05, ***p* < 0.01, ****p* < 0.001, *****p* < 0.0001, ns= not significant.
