# Supplementary material for: Regulation of acute myocardial infarction by CircTMCC1 through mitochondrial dysfunction and AMPK/mTOR-driven M1 macrophage polarization: role in QFR assessment
Source: Cell Death Discov. 2026 Jul 6;12:386. doi: 10.1038/s41420-026-03213-9 (PMC13620124; doi:10.1038/s41420-026-03213-9)
Supplement: Supplementary file 4 [file 41420_2026_3213_MOESM5_ESM.docx]

Supplementary Table 1. The sequences of siRNAs.

| siRNA name | Sense (5'-3') | Antisense (5'-3') |
| --- | --- | --- |
| Si-circTMCC1-1 | GAACUGCGGAGCGGCUUUUTT | AAAAGCCGCUCCGCAGUUCTT |
| Si-circTMCC1-2 | CGGAGCGGCUUUUCCUAAATT | UUUAGGAAAAGCCGCUCCGTT |
| Si-annexin A1 | GCCUUGCAUAAGGCCAUAATT | UUAUGGCCUUAUGCAAGGCTT |
