# Supplementary material for: Regulation of acute myocardial infarction by CircTMCC1 through mitochondrial dysfunction and AMPK/mTOR-driven M1 macrophage polarization: role in QFR assessment
Source: Cell Death Discov. 2026 Jul 6;12:386. doi: 10.1038/s41420-026-03213-9 (PMC13620124; doi:10.1038/s41420-026-03213-9)
Supplement: Supplementary file 5 [file 41420_2026_3213_MOESM6_ESM.docx]

Supplementary Table 2. Primers used in RT-PCR.

| Primer name | Forward primer (5'->3') | Reverse primer (5'->3') |
| --- | --- | --- |
| Has_circTMCC1 | AGGGAACTGCGGAGCGGCTT | TGCCTATCTTCATCACTCTTCCT |
| Has_Annexin A1 | GTGAAGTGCGCCACAAGCAA | GGCGAGTTCCAACACCTTTCA |
| hsa_GAPDH | AAGGTCGGAGTCAACGGATT | ATGGAATTTGCCATGGGTGG |
| hsa_U6 | CTCGCTTCGGCAGCACA | AACGCTTCACGAATTTGCGT |
| mmu-circTMCC1 | GGCAACTGCAGAACGGCT | GCTGAGAGATGGAGGCTT |
| mmu_GAPDH | GTGTTCCTACCCCCAATGTG | CATCGAAGGTGGAAGAGTGG |
| TMCC1 | AAGGGAAGCTATTGCTGGGA | CGAAGGCTTCATGCCTATCTTC |
| Has_TNF-α | GCCTCTTCTCCTTCCTGATCG | TCGAGAAGATGATCTGACTGCC |
| Has_IL-6 | TGCAATAACCACCCCTGACC | AGCTGCGCAGAATGAGATGA |
| Has_CCL2 | AGAATCACCAGCAGCAAGTG | TCCATGGAATCCTGAACCCA |
