# Supplementary material for: Regulation of acute myocardial infarction by CircTMCC1 through mitochondrial dysfunction and AMPK/mTOR-driven M1 macrophage polarization: role in QFR assessment
Source: Cell Death Discov. 2026 Jul 6;12:386. doi: 10.1038/s41420-026-03213-9 (PMC13620124; doi:10.1038/s41420-026-03213-9)
Supplement: Supplementary file 6 [file 41420_2026_3213_MOESM7_ESM.docx]

Supplementary Table 3. The baseline characteristics of the subjects grouped according to CAD status.

| Variables | CAD patients (n = 130) | Controls (n = 43) | Statistical  parameter | *p*-value |
| --- | --- | --- | --- | --- |
| Age (years) | 64.000±11.190 | 57.605±10.981 | 3.264 | 0.001 |
| Gender (male/female) | 86/44 | 23/20 | 2.224 | 0.136 |
| Smoke (yes/no) | 53/77 | 11/32 | 3.198 | 0.074 |
| Drink (yes/no) | 41/89 | 9/34 | 1.770 | 0.183 |
| HTN (yes/no) | 81/49 | 13/30 | 13.398 | 0.000 |
| TC (mmol/L) | 3.942±1.028 | 4.226±1.153 | -1.495 | 0.137 |
| TG (mmol/L) | 1.205(0.980-1.748) | 1.220(1.040-2.110) | -0.787 | 0.431 |
| HDL-C (mmol/L) | 1.033±0.217 | 1.088±0.251 | -1.354 | 0.177 |
| LDL-C (mmol/L) | 2.462±0.818 | 2.675±0.911 | -1.413 | 0.159 |
| Bun (mmol/L) | 5.590(4.685-6.865) | 5.690(4.570-6.660) | -0.081 | 0.936 |
| Ua (μmol/L) | 356.830±100.992 | 343.760±91.642 | 0.737 | 0.462 |
| Scr (μmol/L) | 72.042±20.699 | 68.614±14.531 | 0.995 | 0.321 |
| Nt-pro-BNP (pg/mL) | 142.900(49.055-564.150) | 82.520(46.100-146.100) | -1.383 | 0.167 |
| CircTMCC1 | 2.075(0.962-4.215) | 0.897(0.659-1.255) | -4.382 | <0.001 |
| Gensini Score | 41.500(20.000-92.000) | 0(0-5) | -9.513 | <0.001 |

Data are summarized by mean±standard deviation for normal variables, 50th (25th/75th) percentiles for non-normal variables and N1/N2 for binary variables. The value of each circRNA means the relative amount calculated by 2^−ΔΔct^ method. CAD: coronary artery disease; HTN: hypertension; TC: Total cholesterol; TG: Triglyceride; HDL-C: High-density lipoprotein cholesterol; LDL-C: Low-density lipoprotein cholesterol; Bun: blood urea nitrogen; Ua: Uric Acid; Scr: serum creatinine; Nt-pro-BNP: N-terminal pro-brain natriuretic peptide.
