# Supplementary material for: Regulation of acute myocardial infarction by CircTMCC1 through mitochondrial dysfunction and AMPK/mTOR-driven M1 macrophage polarization: role in QFR assessment
Source: Cell Death Discov. 2026 Jul 6;12:386. doi: 10.1038/s41420-026-03213-9 (PMC13620124; doi:10.1038/s41420-026-03213-9)
Supplement: Supplementary file 7 [file 41420_2026_3213_MOESM8_ESM.docx]

Supplementary Table 4. Univariate logistic regression analyses for the predictors of CAD prevalence.

| Variables | *β*-coefficient | *OR* (95%*CI*) | *p*-value |
| --- | --- | --- | --- |
| circTMCC1 | 0.533 | 1.704(1.248-2.326) | 0.001 |
| Age (years) | 0.052 | 1.053(1.019-1.088) | 0.002 |
| Gender (male/female) | -0.53 | 0.588(0.292-1.186) | 0.138 |
| Smoke (yes/no) | 0.694 | 2.002(0.928-4.321) | 0.077 |
| Drink (yes/no) | 0.554 | 1.74(0.765-3.962) | 0.187 |
| HTN (yes/no) | 1.339 | 3.815(1.818-8.005) | <0.001 |
| TC (mmol/L) | -0.248 | 0.781(0.563-1.084) | 0.139 |
| TG (mmol/L) | -0.054 | 0.947(0.632-1.419) | 0.792 |
| HDL-C (mmol/L) | -1.057 | 0.347(0.074-1.621) | 0.178 |
| LDL-C (mmol/L) | -0.293 | 0.746(0.495-1.124) | 0.161 |
| Bun (mmol/L) | 0.033 | 1.033(0.915-1.167) | 0.598 |
| Ua (μmol/L) | 0.001 | 1.001(0.998-) | 0.460 |
| Scr (μmol/L) | 0.010 | 1.010(0.990-1.030) | 0.320 |
| Nt-pro-BNP (pg/mL) | 0.001 | 1.001(1.000-1.001) | 0.184 |
| LADQFR | -9.747 | 0(0-0.022) | 0.001 |
| LADRMR (mmHg·s/cm) | -0.283 | 0.788(0.682-0.911) | 0.001 |
| LADHMR (mmHg·s/cm) | -0.969 | 0.380(0.230-0.625) | <0.001 |
| LCXQFR | -31.466 | 0 | <0.001 |
| LCXRMR (mmHg·s/cm) | -0.190 | 0.827(0.722-0.947) | 0.006 |
| LCXHMR (mmHg·s/cm) | -0.972 | 0.378(0.211-0.677) | 0.001 |
| RCAQFR | -61.215 | 0 | <0.001 |
| RCARMR (mmHg·s/cm) | -0.336 | 0.715(0.589-0.867) | 0.001 |
| RCAHMR (mmHg·s/cm) | -1.406 | 0.244(0.118-0.505) | <0.001 |
| Gensini Score | 0.455 | 1.576(1.301-1.910) | <0.001 |

*β*-coefficient: regression coefficient; *OR*: odd ratio; *CI*: confidence interval; HTN: hypertension; TC: Total cholesterol; TG: Triglyceride; HDL-C: High-density lipoprotein cholesterol; LDL-C: Low-density lipoprotein cholesterol; Bun: blood urea nitrogen; Ua: Uric Acid; Scr: serum creatinine; Nt-pro-BNP: N-terminal pro-brain natriuretic peptide; LAD: left anterior descending artery; LCX: left circumflex artery; RCA: right coronary artery; QFR: Quantitative Flow Ratio; RMR: Resting Microvascular Resistance; HMR: Simulated Hyperemic Microvascular Resistance; circTMCC1: the relative expression level calculated by 2^-ΔΔct^ method.
