# Supplementary material for: Regulation of acute myocardial infarction by CircTMCC1 through mitochondrial dysfunction and AMPK/mTOR-driven M1 macrophage polarization: role in QFR assessment
Source: Cell Death Discov. 2026 Jul 6;12:386. doi: 10.1038/s41420-026-03213-9 (PMC13620124; doi:10.1038/s41420-026-03213-9)
Supplement: Supplementary file 8 [file 41420_2026_3213_MOESM9_ESM.docx]

Supplementary Table5. Multivariate logistic regression analyses for the predictors of CAD prevalence. Variables included in the multivariate model: age, Drink, HTN, circTMCC1.

| Variables | *β*-coefficient | *OR* (95%*CI*) | *p*-value |
| --- | --- | --- | --- |
| Age (years) | 0.046 | 1.047(1.006,1.089) | 0.037 |
| FBG (mmol/L) | 0.647 | 1.911(1.055-3.461) | 0.033 |
| Gender(male/female) | 1.017 | 2.765(1.121-6.819) | 0.027 |
| HTN (yes/no) | -1.395 | 0.248(0.104-0.594) | 0.002 |
| hsa_circ_0067323 | 0.652 | 1.919(1.325-2.779) | 0.001 |

*β*-coefficient: regression coefficient; *OR*: odd ratio; *CI*: confidence interval; HTN: hypertension; FBG: Fasting Blood Glucose; hsa_circ_0067323: the relative expression level calculated by 2^-ΔΔct^ method.
